# Sustained *in situ* exosome delivery reprograms the diabetic wound microenvironment to unlock immuno-angiogenic repair

**DOI:** 10.1093/rb/rbag116

**Published:** 2026-07-13

**Authors:** Ning Xu, Lili Wang, Yingliang Wei, Pingzhou Zou, Suyang Xu, Chenjun Liu, Bohao Yin, Junjie Guan, Zhiyuan Fan, Shengting Wu, Huiyong Dai, Sa Song, Xin Qi, Wei Zhang

**Affiliations:** Department of Orthopaedic Surgery, Shanghai Sixth People’s Hospital Affiliated to Shanghai Jiao Tong University School of Medicine, Shanghai 200233, China; Department of Orthopaedic Surgery, Shanghai Eighth People’s Hospital, Shanghai 200235, China; Department of Gastroenterology, Shanghai Eighth People’s Hospital, Shanghai 200235, China; College of Life Sciences, Shanghai Normal University, Shanghai 200234, China; Department of Gastroenterology, Shanghai Eighth People’s Hospital, Shanghai 200235, China; Department of Gastroenterology, Shanghai Eighth People’s Hospital, Shanghai 200235, China; Department of Orthopaedic Surgery, Shanghai Sixth People’s Hospital Affiliated to Shanghai Jiao Tong University School of Medicine, Shanghai 200233, China; Department of Orthopaedic Surgery, Shanghai Sixth People’s Hospital Affiliated to Shanghai Jiao Tong University School of Medicine, Shanghai 200233, China; Department of Orthopaedic Surgery, Shanghai Sixth People’s Hospital Affiliated to Shanghai Jiao Tong University School of Medicine, Shanghai 200233, China; Department of Orthopaedic Surgery, Shanghai Sixth People’s Hospital Affiliated to Shanghai Jiao Tong University School of Medicine, Shanghai 200233, China; Department of Orthopaedic Surgery, Shanghai Sixth People’s Hospital Affiliated to Shanghai Jiao Tong University School of Medicine, Shanghai 200233, China; Department of Orthopaedic Surgery, Shanghai Sixth People’s Hospital Affiliated to Shanghai Jiao Tong University School of Medicine, Shanghai 200233, China; Department of Orthopaedic Surgery, Shanghai Sixth People’s Hospital Affiliated to Shanghai Jiao Tong University School of Medicine, Shanghai 200233, China; Department of Orthopaedic Surgery, Shanghai Sixth People’s Hospital Affiliated to Shanghai Jiao Tong University School of Medicine, Shanghai 200233, China; Department of Orthopaedic Surgery, Shanghai Sixth People’s Hospital Affiliated to Shanghai Jiao Tong University School of Medicine, Shanghai 200233, China

**Keywords:** diabetic wound, diabetes, wound repair, nano-repair medicine, exosomes, nanofibrous scaffold

## Abstract

Chronic diabetic wounds persist because inflammation, oxidative stress and impaired angiogenesis create a hostile microenvironment. We developed an exosome-functionalized, aligned poly(lactic-co-glycolic acid)/gelatin electrospun dressing (EE@PG) that serves as a local depot for sustained delivery of endothelial progenitor cell-derived exosomes. EE@PG scavenged hydroxyl and superoxide radicals and showed enzyme-mimetic antioxidant activity. Under lipopolysaccharide stimulation, EE@PG lowered intracellular reactive oxygen species, restored mitochondrial membrane potential and promoted RAW264.7 macrophage polarization toward a reparative phenotype while improving human umbilical vein endothelial cell viability and tube formation. In a splinted full-thickness STZ-induced diabetic rat model, EE@PG accelerated wound closure, enhanced re-epithelialization and collagen remodeling and increased neovascularization and vessel maturation. Transcriptomic analysis supported a shift toward immune resolution and tissue repair programs. Hemolysis, serum chemistry and organ histology indicated favorable biosafety. This work highlights a generalizable drug-delivering nanofibrous dressing that couples sustained exosome signaling with aligned structural cues for chronic wound management in clinically relevant settings.

## Graphical Abstract

**Graphical Abstract:**
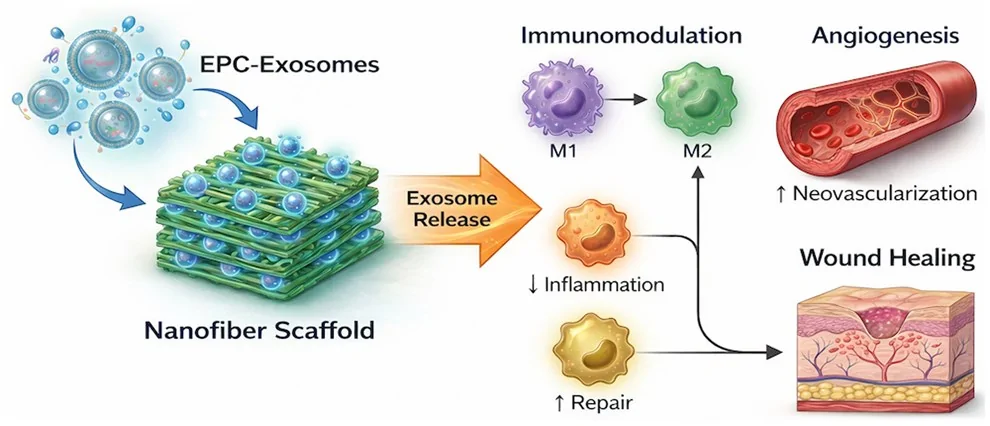

## Introduction

Chronic diabetic wounds (DWs) are a global clinical challenge associated with high morbidity and mortality, driven by persistent inflammation, oxidative stress and defective neovascularization that together stall re-epithelialization and matrix remodeling [1, 2]. Normal wound repair proceeds through hemostasis/inflammation, proliferation and remodeling, each of which requires tightly regulated immunomodulation and epithelialization [3]. In hyperglycemic settings, dysregulated immune signaling and microvascular dysfunction derail these phases, sustaining a non-resolving inflammatory milieu and delaying closure [4]. Growing interest has focused on bioactive macromolecules—particularly extracellular vesicles (EVs)/exosomes, growth factors and cytokines—to therapeutically reprogram the wound microenvironment and enhance repair efficiency [5–8]. In particular, biomaterial-assisted exosome delivery systems, such as controlled-release hydrogel platforms, have demonstrated improved stability and therapeutic efficacy in cutaneous repair [9]. Immune cells and angiogenic mediators—most notably platelet-derived growth factor and vascular endothelial growth factor (VEGF)—coordinate the formation of granulation tissue and the assembly of the vascular network during repair [10]. As inflammation resolves, macrophages transition from an M1-like to an M2-like phenotype and secrete pro-angiogenic factors that promote endothelial activation and tissue regeneration [11, 12]. Therapeutically biasing this balance toward timely M2 polarization has emerged as a strategy to accelerate neovascularization and improve DW healing outcomes [13].

Despite advances in skin grafting, negative-pressure wound therapy and pharmacological adjuncts, clinical outcomes remain suboptimal due to cost, prolonged hospitalization, recurrence and risk of amputation [14]. EV-based approaches—particularly exosomes derived from mesenchymal stem cells and endothelial progenitor cells (EPCs)—have shown promise by delivering immunomodulatory and pro-angiogenic cargo that enhances revascularization and tissue regeneration [15, 16]. EPC-derived exosomes (EPC-Exos) can promote endothelial repair and tissue reconstruction and have been implicated in driving M2 polarization, thereby coupling immune resolution with angiogenesis [17–19]. However, free exosomes are rapidly cleared *in vivo*, limiting their residence time and therapeutic durability [20, 21], which underscores the need for controlled and sustained delivery systems. Conventional dressings (e.g., degreased/oil gauze) provide limited bioactivity and often adhere to wound beds, risking secondary injury upon removal [22, 23]. Next-generation polymeric dressings, including collagen, chitosan, polycaprolactone, poly(lactic-co-glycolic acid) (PLGA), and polyvinyl alcohol, offer tunable mechanics and degradation properties suitable for soft-tissue repair [24–26]. Among these, PLGA is widely used for fibrous scaffolds due to its mechanical robustness, controllable degradation and established biocompatibility profiles; however, the lack of cell-recognition motifs and inherent hydrophobicity can impede cell adhesion and biointegration [27, 28].

Electrospinning yields nanofibrous scaffolds with high surface area, interconnected porosity and extracellular matrix (ECM)-mimetic topography, which support cell adhesion and guide tissue regeneration [29–33]. Aligned fiber architectures can provide contact guidance for cells and facilitate directional biomolecule distribution, and composite designs enable the incorporation of therapeutic agents. Prior studies demonstrate that electrospun systems can deliver drugs or biologics to enhance skin repair, highlighting the translational potential of multi-component nanofibers [34–36]. To overcome PLGA’s limited hydrophilicity and lack of biological ligands, blending with gelatin introduces natural cell-recognition sites, improving wettability, moisture handling and cell-material interactions that are conducive to wound repair [37]. Therefore, a PLGA/gelatin composite nanofibrous scaffold represents a suitable structural platform for DW dressing design. However, structural support alone is insufficient to effectively reverse the persistent inflammatory dysregulation and impaired angiogenesis that characterize DWs. In parallel, incorporating bioactive macromolecules—such as EPC-Exos—into the nanofiber matrix or onto fiber surfaces may enable sustained local release while simultaneously modulating immune responses and angiogenesis, potentially synchronizing macrophage reprogramming and endothelial activation within the DW microenvironment.

In this study, we propose an integrated strategy to address the hostile microenvironment of diabetic chronic wounds by combining structural guidance with bioactive signal delivery. An aligned PLGA/gelatin nanofibrous substrate was fabricated by electrospinning to mimic key ECM features and provide a conformal dressing interface, and it was subsequently endowed with exosome-based bioactivity through a mild functionalization approach that enables local retention and sustained release (Figure 1). Distinct from conventional passive dressings or bolus administration, the key innovation of this work lies in coupling a sustained EV delivery modality with an ECM–mimetic, aligned fibrous architecture, thereby targeting two central barriers to DW repair: persistent inflammatory dysregulation and insufficient vascular regeneration. By reshaping the wound milieu toward a pro-repair immune profile while simultaneously enhancing angiogenesis and vascular maturation, this platform promotes more effective and higher-quality tissue regeneration. Collectively, our findings establish a broadly applicable design concept for exosome-enabled, drug-delivering nanofibrous dressings and provide mechanistic insight with potential translational relevance for the treatment of refractory DWs.

**Figure 1:**
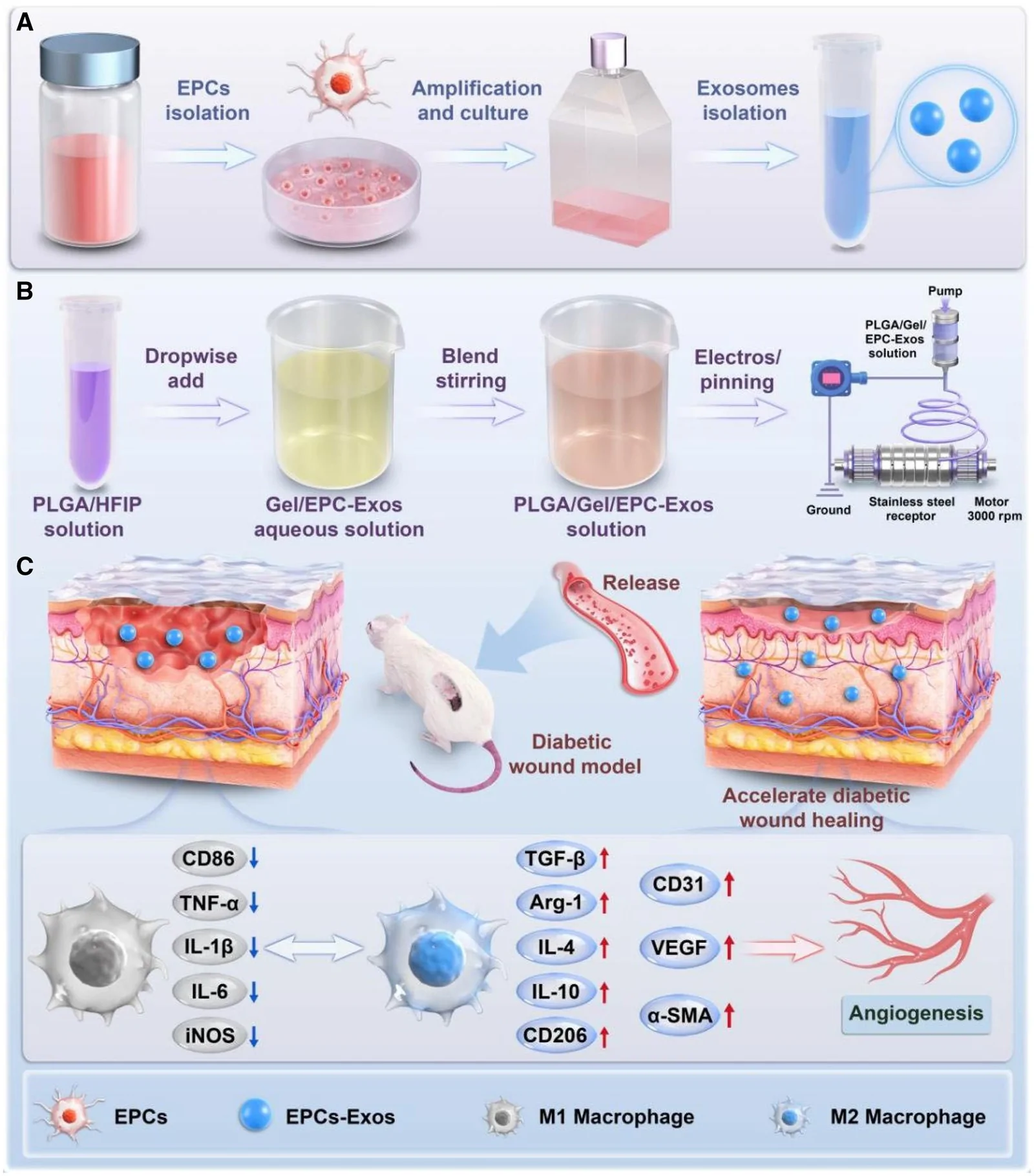
Overall schematic. (**A**) Schematic illustration of the preparation of EPC-Exos (EE). (**B**) Fabrication of EE@PG nanofibrous mats by electrospinning a PLGA/gelatin (PG) solution followed by EE loading. (**C**) Proposed therapeutic application of EE@PG in diabetic wound healing through coordinated immunomodulation and pro-angiogenic microenvironment remodeling.

## Materials and methods

### Materials, reagents and consumables

PLGA (M_n_ ≈ 100 kDa, LA/GA = 50/50, inherent viscosity 2.4 dL/g; Jinan Dai Gang Biotechnology, China) and gelatin (Type B, porcine skin; Sigma-Aldrich) were used to fabricate electrospun nanofibrous mats. 1,1,1,3,3,3-Hexafluoro-2-propanol (HFIP, 99.5%; Shanghai Darui, China) and phosphate-buffered saline (PBS, pH 7.4) were used as received. For cell culture, high glucose Dulbecco’s Modified Eagle Medium, EGM-2 MV supplements, penicillin/streptomycin, trypsin, lipopolysaccharide (LPS) and fetal bovine serum (FBS) were obtained from Thermo Fisher Scientific; EV-depleted FBS was prepared by ultracentrifugation (100 000 × g, 18 h) prior to use. Cell Counting Kit-8 (CCK-8) Assay Kit, Hydrogen Peroxide (H_2_O_2_) Assay Kit, and Superoxide Dismutase (SOD) Assay Kit (WST-8 method) were purchased from Beyotime (Shanghai, China). Sterility and endotoxin were assessed using a limulus amebocyte lysate (LAL) kit and sterility culture media (Sigma-Aldrich).

### Ethics approval

Human EPCs were derived from umbilical cord blood under institutional review board approval with informed consent. All animal experiments complied with ARRIVE 2.0 guidelines and were approved by the Institutional Animal Care and Use Committee (IACUC) of Shanghai Sixth People’s Hospital (No. 2019-0386).

### EPC culture and exosome isolation

#### EPC culture

Human EPCs were isolated from umbilical cord blood and expanded in EGM-2 MV supplemented with EV-depleted FBS at 37°C and 5% CO_2_. Cell authentication (short tandem repeat profiling) and mycoplasma testing (MycoAlert^®^) were performed routinely. EPC identity was confirmed by Dil-acLDL uptake (10 µg/mL, 4 h) and FITC-UEA-1 binding (10 µg/mL, 1 h), along with flow cytometry/immunostaining positivity for CD34/VEGFR2/CD133 (>90%).

#### Exosome isolation

Conditioned medium was sequentially cleared (300 × g 15 min; 2000 × g 20 min, 4°C), filtered (0.22 µm), and subjected to differential ultracentrifugation with a 30% sucrose/D_2_O cushion (100 000 × g, 2 h, 4°C). Pellets were washed in PBS and concentrated via ultrafiltration (4,000 × g, 2 × 20 min), then resuspended in PBS containing 0.1% bovine serum albumin (BSA). Exosomes were aliquoted and stored at −80°C with ≤1 freeze–thaw cycle. Exosome dose was reported as particle number [nanoparticle tracking analysis (NTA)] and protein amount [bicinchoninic acid (BCA)].

### Exosome characterization and quality control

#### Nanoparticle tracking analysis

Particle size distribution and concentration were measured using a NanoSight NS300 system (Malvern Panalytical, UK) equipped with a 488-nm laser and an sCMOS camera. Exosome suspensions were diluted in sterile PBS to achieve a final particle concentration of ∼1 × 10^8^–1 × 10^9^ particles/mL for optimal tracking. For each sample, three 60-s videos were recorded (frame rate 30 fps, 1800 frames per video; total 5400 frames per sample) under consistent settings (camera level 13–15, detection threshold 5–7, temperature controlled at 22–25°C). Data were analyzed using NTA software (Malvern Panalytical), reporting mode size and D10/D50/D90 values as mean ± SD across the three captures.

#### Transmission electron microscopy

Exosome morphology was examined using a transmission electron microscope (TEM; JEOL JEM-2100, Japan) operated at 200 kV. Briefly, 10 µL of exosome suspension was loaded onto a Formvar/carbon-coated copper grid (200 mesh) for 10 min, blotted, and negatively stained with 2% (w/v) phosphotungstic acid (pH 7.0) for 2 min. After air-drying, images were acquired at multiple magnifications with consistent acquisition parameters.

#### Western blotting

Exosome preparations and parental EPC lysates were analyzed for canonical exosomal markers. Exosomal protein concentration was quantified using a BCA assay, and 20 µg total protein per lane was loaded onto 10% SDS–PAGE gels, followed by transfer to 0.22-µm PVDF membranes. Membranes were blocked with 5% (w/v) non-fat milk in TBST for 1 h at room temperature and incubated overnight at 4°C with primary antibodies against CD9, CD63, CD81 (each 1:1000) and a negative marker calnexin (1:1000). After washing, Horseradish peroxidase (HRP)-conjugated secondary antibodies were applied (1:5000, 1 h, room temperature). Protein bands were visualized using ECL substrate and imaged with a ChemiDoc MP system (Bio-Rad, USA). To ensure signals remained within the linear range, multiple exposure times were collected (10, 30 and 60 s), and the exposure used for quantification was selected based on non-saturated bands. To evaluate whether released EPC-Exos retained the expression of characteristic exosomal markers after scaffold loading, EPC-Exos released from EE@PG were collected at the 72 h time point and compared with freshly isolated EPC-Exos. Western blotting (WB) for CD9 and CD63 was performed to assess exosomal marker expression. Densitometry (when performed) was analyzed using ImageJ.

#### *In vitro* release kinetics of EPC-Exos from EE@PG

To evaluate the *in vitro* release behavior of EPC-Exos from the EE@PG nanofibrous membrane, EE@PG samples with a predetermined exosome-loading amount were immersed in 2 mL of PBS (pH 7.4) and incubated at 37°C under gentle shaking. At predetermined time points (1, 3, 5, 7, 14 and 21 days), the entire release medium was collected and replaced with an equal volume of fresh PBS to maintain sink conditions. The collected supernatants were stored at 4°C for subsequent analysis. The amount of released EPC-Exos at each time point was quantified using a BCA protein assay kit according to the manufacturer’s instructions. Briefly, the protein concentration in each collected release sample was measured, and the cumulative release percentage of EPC-Exos was calculated based on the total initially loaded exosome amount. The cumulative release was calculated using the following equation:


$$\mathrm{Cumulative}\;\mathrm{release}\;(\%)=\frac{\sum M_{t}}{M_{0}}\times 100\%,$$

where *M_t_* represents the amount of EPC-Exos released at each sampling time point, and *M*_0_ represents the total amount of EPC-Exos initially loaded onto the nanofibrous membrane.

### Fabrication of aligned PLGA/gelatin (PG) nanofibers and exosome loading (EE@PG)

#### Electrospinning

PLGA was dissolved in HFIP at 10% (w/v). Gelatin was prepared at 10% (w/v) in water. Immediately prior to spinning, solutions were mixed at a PLGA: gelatin mass ratio of 70:30. To improve miscibility, formic acid (5% v/v) was added to obtain a homogeneous electrospinning dope (viscosity: 1000 mPa·s). The solution was electrospun onto a high-speed rotating mandrel (diameter 20 cm) to obtain aligned fibers. Electrospinning parameters were voltage 15 kV, flow rate 1.0 mL/h, tip-to-collector distance 15 cm, collector speed 3000 rpm, ambient temperature 22°C and relative humidity 15%. Mats were vacuum-dried for 48 h to remove residual solvent.

#### Sterilization

For *in vitro* and *in vivo* experiments, patches were sterilized by γ-irradiation or ethylene oxide (EO). Residual EO was removed according to the manufacturer’s guidance. UV/ethanol treatment was not used for final implants.

#### Exosome loading (post-spinning adsorption)

To avoid solvent exposure, EPC-Exos were loaded after electrospinning. PG discs were incubated with EPC-Exos at 1 × 10^10^ particles/cm^2^ in PBS containing 0.1% BSA for 12 h at 4°C, followed by gentle rinsing to remove unbound exosomes, generating EE@PG. Loading efficiency was calculated by mass balance (input minus supernatant and wash fractions). The amount of exosomes loaded onto the scaffold was calculated by subtracting the residual amount in the supernatant and wash fractions from the initial input amount. The loading efficiency was calculated according to the following equation:


$$\mathrm{Loading}\;\mathrm{efficiency}\;(\%)=\frac{M_{0}-(M_{S}+M_{w})}{M_{0}}\times 100\%,$$

where *M_0_* is the initial amount of EPC-Exos added, *M*_S_ is the amount remaining in the supernatant after loading, and *M*_w_ is the amount recovered in the wash fractions. Exosome localization on/within fibers could be verified by immunogold TEM.

### Antioxidant and reactive oxygen species-scavenging assays

#### ESR detection of •OH scavenging

Hydroxyl radicals were generated using a Fenton reaction (Fe^2+^/H_2_O_2_). The final reaction mixture contained DMPO (50 mM), FeSO_4_ (100 µM), and H_2_O_2_ (1 mM). PG or EE@PG extracts (equivalent to 2 mg/mL scaffold) were added. ESR spectra were acquired at 1 min, and •OH levels were quantified using the DMPO-OH quartet amplitude normalized to the control.

#### ESR detection of •O_2_^−^ scavenging

Superoxide was generated using riboflavin (10 mM in methanol) under light exposure. DMPO (50 mM) was added, and ESR signals were collected in the presence of increasing concentrations of scaffold extracts.

#### CAT-like and SOD-like activities

Residual H_2_O_2_ was quantified using an H_2_O_2_ assay kit at 560 nm (xylenol orange method), and SOD-like activity was assessed using a WST-8 assay at 450 nm, following manufacturer instructions. Results were normalized to scaffold mass or surface area and reported as mean ± SD (*n* = 3).

### Morphological and physicochemical characterization of nanofibers

#### SEM/TEM

Nanofiber mats were sputter-coated with gold (5 mA, 35 s) and imaged using SEM (Phenom XL, 10 kV). Fiber diameter (≥200 fibers per batch) and alignment/orientation distributions were quantified using ImageJ/OrientationJ. TEM (JEOL JEM-2100) was used to assess morphology.

#### Water contact angle

Static contact angles were measured using a goniometer (JC-2000D-2A) with 20-µL droplets; ≥5 locations per sample were tested.

#### Mechanical properties and degradation

Uniaxial tensile testing was performed under dry and hydrated conditions to obtain Young’s modulus, ultimate tensile strength and strain at break. *In vitro* degradation was evaluated by incubating samples in PBS and measuring mass loss and thickness change at predetermined time points.

### *In vitro* cytocompatibility

Sterile PG and EE@PG discs (14 mm) were fixed in 24-well plates using sterile stainless-steel rings and pre-wetted overnight in complete medium. Human umbilical vein endothelial cells (HUVECs) or NIH-3T3 cells were seeded at 1 × 10^5^ cells/well and cultured at 37°C with 5% CO_2_; medium was replaced every 2 days. At days 1, 3 and 5, medium was replaced with CCK-8 working solution (1:10 dilution in fresh medium; 400 µL/well) and incubated for 2 h. Absorbance was measured at 450 nm. Cell viability/proliferation was normalized to day-1 PG controls.

### Cell attachment and live/dead staining

HUVECs (1 × 10^5^/well) were seeded on PG or EE@PG discs (14 mm) and cultured for 24 h. After PBS washing, cells were stained with calcein-AM and ethidium homodimer-1 following kit instructions and imaged using a fluorescence microscope (Nikon TS100) with consistent exposure settings. Attachment density and viability were quantified from ≥3 random fields per sample by a blinded analyst.

Control, LPS, PG and EE@PG groups were included, with *n* = 3 independent replicates per group. LPS concentration and stimulation duration were consistent with macrophage experiments.

### Macrophage polarization and oxidative stress assays

For inflammatory challenge experiments, macrophages were first stimulated with LPS (1.0 µg/mL) for 6 h, followed by replacement with fresh culture medium and treatment/coculture with the corresponding materials, with a total treatment duration of 24 h. Cells were collected after 24 h for the following analysis.

#### Flow cytometry

Cells were blocked with anti-CD16/32 and stained with CCR7/CD197 (M1) and CD206 (M2) antibodies (1:100). M1/M2 percentages were calculated.

#### Quantitative real-time polymerase chain reaction

iNOS/Arg-1 and cytokine genes were quantified (see “*In vitro* angiogenesis assay” for polymerase chain reaction details).

#### Enzyme-linked immunosorbent assay

Tumor necrosis factor (TNF)-α, interleukin (IL)-6 and IL-10 in supernatants were measured using commercial kits.

#### Intracellular reactive oxygen species

2′,7′-Dichlorodihydrofluorescein diacetate (DCFH-DA) staining was performed and analyzed by fluorescence imaging and flow cytometry.

#### Mitochondrial membrane potential (ΔΨm)

JC-1 staining was performed and quantified as the red/green fluorescence ratio; mitochondrial staining with MitoTracker was conducted as supportive imaging.

Experimental groups. Control, LPS, PG and EE@PG groups were used for macrophage polarization readouts; for reactive oxygen species (ROS)/ΔΨm assays and tube formation under inflammatory conditions, groups were Control, LPS, LPS+PG and LPS+EE@PG.

### *In vitro* angiogenesis assay

Matrigel was thawed on ice and used to coat pre-chilled 96-well plates (50 µL/well). HUVECs were seeded at 1 × 10^4^ cells/well in endothelial basal medium supplemented with 2% (v/v) FBS (reduced-serum condition) and treated according to group assignment: Control, LPS, LPS+PG and LPS+EE@PG. After 6 h of incubation at 37°C, tube structures were imaged under an inverted microscope. To evaluate whether EPC-Exos released from EE@PG retained pro-angiogenic activity, HUVECs were seeded onto Matrigel-coated 96-well plates and treated, under otherwise identical conditions, with either freshly isolated EPC-Exos or EPC-Exos released from EE@PG at the 72 h time point. Total tube length, number of junctions and meshes were quantified using ImageJ (Angiogenesis Analyzer plugin) by blinded analysis (*n* = 3).

### Full-thickness diabetic dorsal wound model and treatments

#### Animals and diabetes induction

Male Sprague–Dawley rats (8–10 weeks old, 250–300 g) were housed under specific pathogen-free conditions at 22 ± 2°C with 50–60% humidity and a 12 h light/dark cycle, with free access to food and water. After overnight fasting, diabetes was induced by a single intraperitoneal injection of streptozotocin (STZ, 60 mg/kg), freshly dissolved in 0.1 M citrate buffer (pH 4.5). Fasting blood glucose levels were measured from tail vein blood using a glucometer at 72 h after STZ injection and monitored weekly thereafter. Rats with fasting blood glucose levels ≥16.7 mmol/L (≈300 mg/dL) on two consecutive measurements were considered diabetic. After 4 weeks of stable hyperglycemia, the diabetic rats were used for the subsequent wound-healing experiments. Briefly, a 10-mm-diameter full-thickness excisional wound was created on the dorsal skin of each rat to establish a diabetic rat dorsal full-thickness wound model.

#### Randomization and blinding

After successful establishment of the DW model, the animals were randomly assigned to the experimental groups according to the predefined study design. Group allocation was completed before treatment administration, and all animals were maintained under the same housing and experimental conditions throughout the study. Wound images were acquired in a standardized manner at predetermined time points to monitor the healing process. Quantitative analyses, including wound area measurement, histological evaluation, and immunostaining quantification, were performed in a blinded manner. Specifically, the investigators responsible for image acquisition and quantitative analysis were unaware of the treatment identities during data collection and analysis. Histological and immunohistochemical evaluations were conducted under identical settings and predefined criteria to minimize observer bias.

#### Wound creation and splinting

Rats were anesthetized with isoflurane (5% induction; 1.5–2% maintenance) and placed on a heating pad. After dorsal hair removal and disinfection (povidone-iodine followed by 70% ethanol), one 10-mm full-thickness excisional wound was created on the dorsum using a sterile biopsy punch. To minimize wound contraction, a silicone splint (inner diameter 10 mm, outer diameter 16 mm, thickness 0.5–1.0 mm) was centered around the wound and fixed with 6-0 nylon interrupted sutures (6–8 stitches). Sterile PG or EE@PG patches (10-mm diameter, ∼200-μm thickness) were applied to fully cover the wound bed.

#### Dressing fixation and replacement

Wounds were covered with a semi-occlusive transparent film dressing (e.g., Tegaderm™) and reinforced with an elastic adhesive bandage. Dressings were changed every 2 days under brief isoflurane anesthesia, and the wound was gently rinsed with sterile saline during each dressing change.

#### Analgesia and monitoring

Buprenorphine (0.05 mg/kg, subcutaneous) was administered every 12 h for 48 h post-surgery. Animals were monitored daily for behavior, wound condition and signs of infection.

#### Macroscopic wound closure

Standardized digital photographs were taken on postoperative days 0, 3, 7 and 14 with a metric ruler included. The wound area was quantified using ImageJ. Wound closure was calculated as:


$$\mathrm{Wound}\;\mathrm{closure}\;(\%)=(1–A_{t}/A_{0})\times 100\%,$$

where *A*_0_ is the wound area at day 0 and *A_t_* is the area at each time point.

#### Tissue harvest and histology

Rats were euthanized at days 3, 7 and 14 (*n* = 3 per group per time point) by overdose of pentobarbital (150 mg/kg, i.p.). Wound tissues including a 2-mm margin of surrounding skin were harvested, fixed in 10% neutral-buffered formalin for 24 h, embedded in paraffin, and sectioned at 5 μm. Hematoxylin and eosin (H&E) and Masson’s trichrome staining were performed to evaluate re-epithelialization, granulation tissue formation and collagen deposition. Quantification was performed on ≥3 random fields per section and ≥3 sections per animal by blinded analysis using predefined ROIs and consistent thresholds.

#### Immunostaining

Immunofluorescence staining for CD31 and alpha smooth muscle actin (α-SMA) was performed on wound sections at days 7 and 14 to evaluate neovascularization and vessel maturation. Macrophage polarization in wound tissues was assessed by co-staining iNOS/Arg-1 and iNOS/CD206 at days 7 and 14. Immunohistochemistry for Ki67, NLRP3, CD86 and TUNEL staining was conducted at days 7 and 14. Briefly, sections underwent antigen retrieval (citrate buffer, pH 6.0), blocking with 5% BSA (1 h), overnight incubation with primary antibodies at 4°C, and incubation with fluorophore-conjugated (for IF) or HRP-conjugated (for IHC) secondary antibodies. Nuclei were counterstained with DAPI for IF, and DAB was used for chromogenic visualization in IHC. Images were acquired under identical exposure settings, and quantification was performed using ImageJ by blinded analysis.

#### Biosafety evaluation

Body weight and fasting blood glucose were recorded weekly. On day 14, major organs (heart, liver, spleen, lung and kidney) were harvested for H&E staining. Serum biochemistry (ALT, AST, BUN and creatinine) was analyzed on day 14 (*n* = 3 per group).

### Quantitative real-time PCR

Total RNA was extracted from RAW264.7 macrophages or wound tissues using TRIzol™ reagent (Thermo Fisher Scientific) according to the manufacturer’s protocol. For wound tissues, samples were snap-frozen in liquid nitrogen, homogenized using a bead mill homogenizer (TissueLyser, Qiagen) in TRIzol and centrifuged to remove debris prior to phase separation. RNA was precipitated with isopropanol, washed with 75% ethanol, and dissolved in RNase-free water. RNA concentration and purity were determined by NanoDrop spectrophotometry, and samples with A260/A280 between 1.8 and 2.1 were used. To eliminate genomic DNA contamination, RNA was treated with gDNA remover (included in the RT kit) or DNase I (Thermo Fisher) as appropriate.

cDNA was synthesized using PrimeScript™ RT Master Mix (TaKaRa, Japan) with 1 µg total RNA in a final volume of 20 µL (37°C for 15 min, 85°C for 5 s). Quantitative PCR was performed using SYBR^®^ Premix Ex Taq™ II (TaKaRa) on a CFX96 Real-Time PCR System (Bio-Rad, USA). Each 20-µL reaction contained 10 µL SYBR mix, 0.4 µL forward primer (10 µM), 0.4 µL reverse primer (10 µM), 2 µL diluted cDNA (equivalent to 10–50 ng RNA input), and 7.2 µL nuclease-free water. The cycling program was 95°C for 30 s, followed by 40 cycles of 95°C for 5 s and 60°C for 30 s. Melt-curve analysis was performed from 65 to 95°C with 0.5°C increments to confirm amplification specificity.

Gene expression was normalized to GAPDH as the internal reference and calculated using the 2^−ΔΔCt^ method. For each sample, qPCR reactions were run in technical duplicates, and experiments included at least three independent biological replicates (*n* = 3). No-template controls and no-reverse-transcription controls (−RT) were included to exclude contamination and genomic DNA amplification. Primer sequences are listed in Table S1.

### Statistical analysis

All data are presented as mean ± SD. Biological replicates were defined as independent cell preparations or individual animals, and technical replicates as repeated measurements of the same sample. For *in vivo* studies, each animal was considered one biological replicate and the unit of analysis. Histological and immunostaining quantification was performed on at least three random fields per section and three sections per animal. Normality and homogeneity of variance were assessed using the Shapiro–Wilk and Levene tests. Comparisons between two groups were analyzed by an unpaired two-tailed Student’s *t*-test, and multiple-group comparisons by one-way or two-way analysis of variance with Tukey’s *post hoc* test. Nonparametric tests were used when required. Exact sample sizes are indicated in the figure legends. *P* < 0.05 was considered statistically significant.

## Results

### EPC-Exos identification and fabrication/characterization of EE@PG

To develop a bioactive dressing capable of addressing the immuno-angiogenic deficits in DWs, EPC-derived exosomes (EPC-Exos; EE) were first isolated from conditioned medium by sequential centrifugation/filtration followed by ultracentrifugation (Figure 2A). TEM revealed intact, cup-shaped EE nanoparticles with a homogeneous morphology (Figure 2B and C). Subsequently, EE was loaded onto pre-fabricated aligned PLGA/gelatin nanofibers via aqueous phase adsorption to construct the EE@PG scaffold (Figure 2D). The loading efficiency of EE on the PG nanofibrous membrane was quantitatively analyzed using a mass balance method. The results showed that the EE loading efficiency of EE@PG was 76.5 ± 0.7%, indicating that the PG nanofibrous scaffold possessed favorable exosome-loading capacity (Table S2).

**Figure 2:**
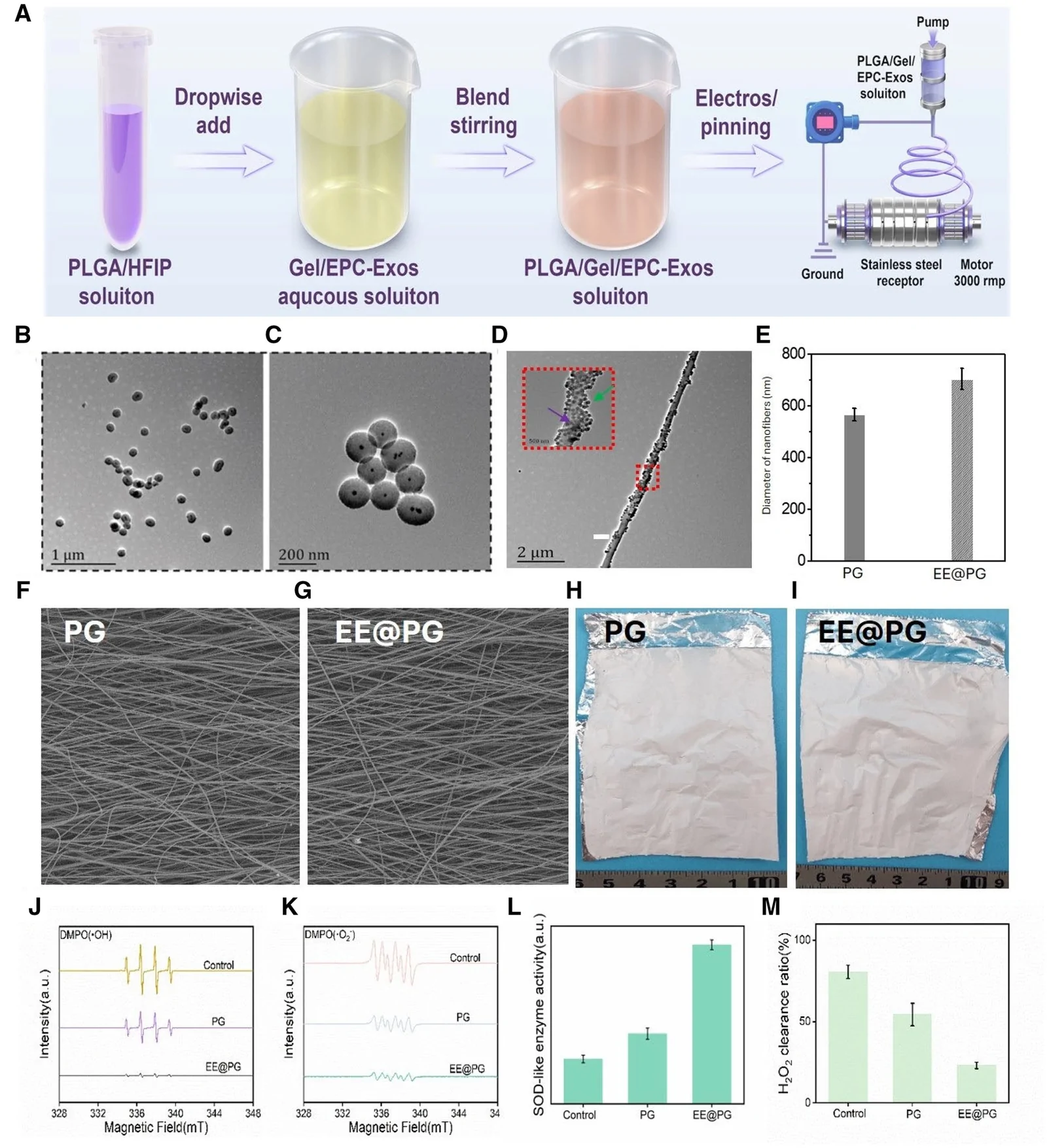
Characterization of EE nanoparticles and EE@PG nanofibrous mats. (**A**) Schematic illustration of EE@PG fabrication. (**B** and **C**) TEM images of purified EE and a higher-magnification view. (**D**) TEM image of an EE@PG nanofiber; purple arrows denote the PG matrix, and green arrows denote EE nanoparticles distributed within the PG matrix. (**E**) Fiber diameter distributions of PG and EE@PG. (**F** and **G**) SEM images of PG and EE@PG nanofibrous mats. (**H** and **I**) Representative photographs of PG and EE@PG mats. (**J** and **K**) Free-radical scavenging capacity assessed by ESR using DMPO as the spin trap: (**J**) •OH and (**K**) •O_2_^−^ scavenging in control, PG and EE@PG groups. (**L** and **M**) Enzyme-mimetic antioxidant activities: (L) SOD-like activity and (M) CAT-like activity and (**M**) SOD-like activity in control, PG and EE@PG groups. Data are presented as mean ± SD (*n* = 3, technical replicate).

To further characterize the exosomal component used in this study, the particle size distribution, marker expression, and release behavior of EPC-Exos from the nanofibrous scaffold were evaluated. As shown in Supplementary Figure S1A, NTA revealed that EE exhibited a relatively concentrated size distribution, mainly ranging from 100 to 200 nm, with a major peak at approximately 130 nm, consistent with the typical size characteristics of exosomes. In addition, the particle concentration reached the order of 10^6^ particles/mL, indicating the successful isolation of a sufficient amount of nanoscale vesicles.

Western blot analysis further confirmed the exosomal identity of the isolated vesicles. As shown in Supplementary Figure S1B, EPC-Exos clearly expressed the characteristic exosomal surface markers CD9, CD63, and CD81, whereas the endoplasmic reticulum marker calnexin was strongly expressed in parental EPCs but was nearly undetectable in EPC-Exos. These results collectively demonstrate that the isolated nanoparticles possessed the typical molecular characteristics of exosomes. In addition, released EPC-Exos retained the expression of characteristic exosomal markers, as confirmed by WB for CD9 and CD63, showing profiles comparable to those of fresh EPC-Exos (Supplementary Figure S2A). These findings support that the released vesicles preserved their basic exosomal identity after scaffold loading and release.

Furthermore, the *in vitro* release behavior of EPC-Exos from the EE@PG nanofibrous scaffold was investigated. As shown in Supplementary Figure S1C, EPC-Exos were continuously released from the scaffold over a period of 21 days. These findings indicate that EE@PG is capable of achieving sustained EPC-Exos release, supporting its potential as a locally controlled exosome delivery platform.

FT-IR analysis of EE@PG displayed characteristic PLGA-related vibrations, including a broad O–H stretching band (∼3450 cm^−1^), C–H stretching (2950–2850 cm^−1^), a prominent ester C=O stretching peak (∼1750 cm^−1^), and C–O–C stretching bands (1180–1080 cm^−1^), supporting successful formation of the composite matrix (Supplementary Figure S3).

Morphological characterization indicated that EE incorporation increased fiber diameter (PG vs EE@PG; Figure 2E). SEM images showed uniform, bead-free fibers for both groups; EE@PG exhibited a slightly roughened surface compared with PG, consistent with exosome incorporation (Figure 2F and G). Macroscopic photographs further confirmed that both mats formed flexible, thin fibrous membranes suitable for wound coverage (Figure 2H and I).

### Antioxidant and free-radical scavenging properties of EE@PG

Given the central role of oxidative stress in impaired DW healing, we next examined the antioxidant capacity of the scaffolds. ESR measurements using DMPO as the spin trap demonstrated that EE@PG markedly reduced signals corresponding to hydroxyl radicals (•OH) and superoxide anions (•O_2_^−^) compared with control and PG (Figure 2J and K). Semi-quantitative ESR analyses further supported the enhanced scavenging capacity of EE@PG for both radical species (Supplementary Figures S4 and S5).

Consistently, EE@PG exhibited stronger CAT-like activity as reflected by increased H_2_O_2_ decomposition (Figure 2L). In addition, EE@PG displayed enhanced SOD-like activity in a concentration-dependent manner (Figure 2M), indicating robust antioxidative potential that may contribute to improved immunomodulation and pro-healing responses in subsequent assays.

### *In vitro* cytocompatibility of EE@PG toward HUVECs and NIH-3T3 cells

The biocompatibility of PG and EE@PG was evaluated using CCK-8 assays. Both HUVECs and NIH-3T3 fibroblasts showed progressive increases in metabolic activity from day 1 to day 5 when cultured on the scaffolds, indicating good cytocompatibility (Figure 3A and B). Notably, EE@PG consistently supported higher cell viability/proliferation than PG across the time course.

**Figure 3:**
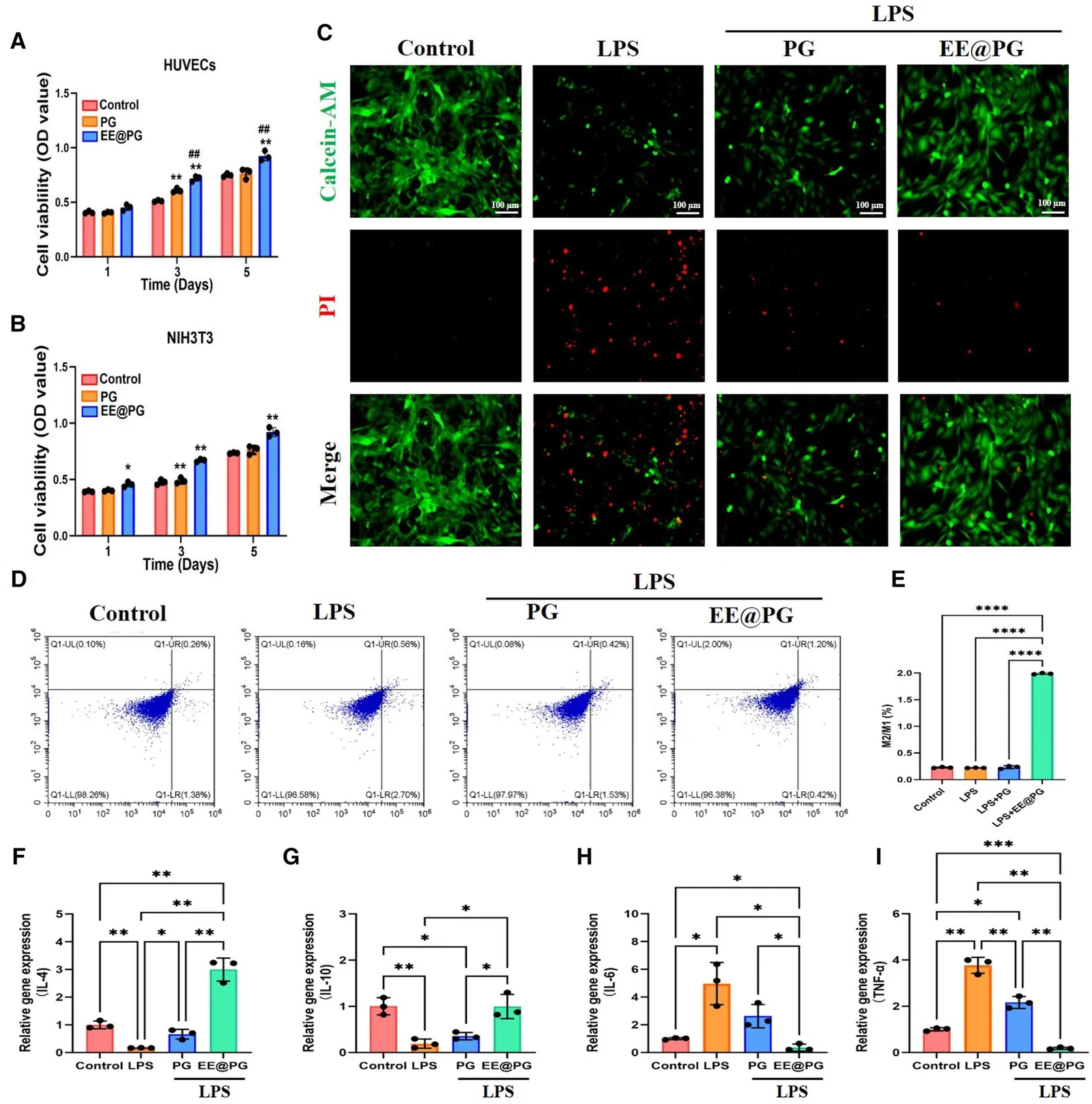
EE@PG enhances cell viability and promotes macrophage M2 polarization under LPS stimulation. (**A** and **B**) Cell proliferation of (**A**) human umbilical vein endothelial cells (HUVECs) and (**B**) NIH3T3 fibroblasts cultured on control, PG and EE@PG nanofibrous mats was evaluated by CCK-8 at days 1, 3 and 5. (**C**) Live/dead staining of HUVECs after 24 h of culture on control, LPS, LPS+PG and LPS+EE@PG groups. (**D**) Polarization of RAW264.7 macrophages was assessed by flow cytometry in control, LPS, LPS+PG and LPS+EE@PG groups. (**E**) Quantification of M2 and M1 macrophage percentages (M2/M1). (**F**–**I**) RT-qPCR analysis of M2-associated genes (*IL-4*, *IL-10*) and M1-associated genes (*TNF-α*, *IL-6*) in control, LPS, LPS+PG and LPS+EE@PG groups. Data are presented as mean ± SD (*n* = 3, technical replicate). *P < 0.05, **P < 0.01, ***P < 0.001, ****P < 0.0001. ## indicates the significant difference between the EE@PG group and the PG (*P* < 0.01), and * represents the significant difference compared with the Control group.

Live/dead staining further confirmed robust HUVEC viability on EE@PG after 24 h (Figure 3C). Semi-quantitative analysis of the live/dead images indicated that LPS challenge reduced viability, whereas PG partially rescued cell survival and EE@PG restored high viability close to the non-LPS condition (Supplementary Figure S6).

Supplementary Figures S7 and S8 provide semi-quantitative analysis of LysoTracker fluorescence in cells incubated with EE@PG-FITC at 2, 4 and 6 h. The LysoTracker signal (and the LysoTracker/EE@PG-FITC co-localization trend) increased in a time-dependent manner, indicating progressive intracellular uptake and subsequent trafficking of EE@PG-FITC toward the endo/lysosomal compartment over the 2–6-h incubation window. These results support that EE@PG-associated cargos can be efficiently internalized and processed through lysosome-related pathways.

### EE@PG promotes macrophage M2 polarization and supports endothelial angiogenic function under inflammatory stress

To test whether EE@PG can reprogram macrophage phenotype under inflammatory conditions, RAW264.7 macrophages were stimulated with LPS and then treated with PG or EE@PG. Flow cytometry showed that LPS+EE@PG increased the proportion of CD206^+^ M2-like macrophages and reduced CCR7^+^ M1-like macrophages compared with LPS and LPS+PG groups (Figure 3D and E). This phenotypic shift was corroborated by qRT-PCR: LPS+EE@PG up-regulated M2-associated genes (*IL-4*, *IL-10*) and down-regulated M1-associated pro-inflammatory genes (*TNF-α*, *IL-6*) relative to LPS controls (Figure 3F–I).

In parallel, angiogenesis-relevant function was assessed using an *in vitro* tube formation assay. Under LPS challenge, HUVECs exhibited impaired network formation, whereas EE@PG improved tubular structure formation compared with LPS and LPS+PG groups (Figure 4A), supporting a pro-angiogenic effect under inflammatory stress. Furthermore, released EPC-Exos exhibited a tube formation-promoting effect comparable to that of fresh EPC-Exos, indicating that the exosomes retained pro-angiogenic bioactivity after scaffold loading and release (Supplementary Figure S2B).

**Figure 4:**
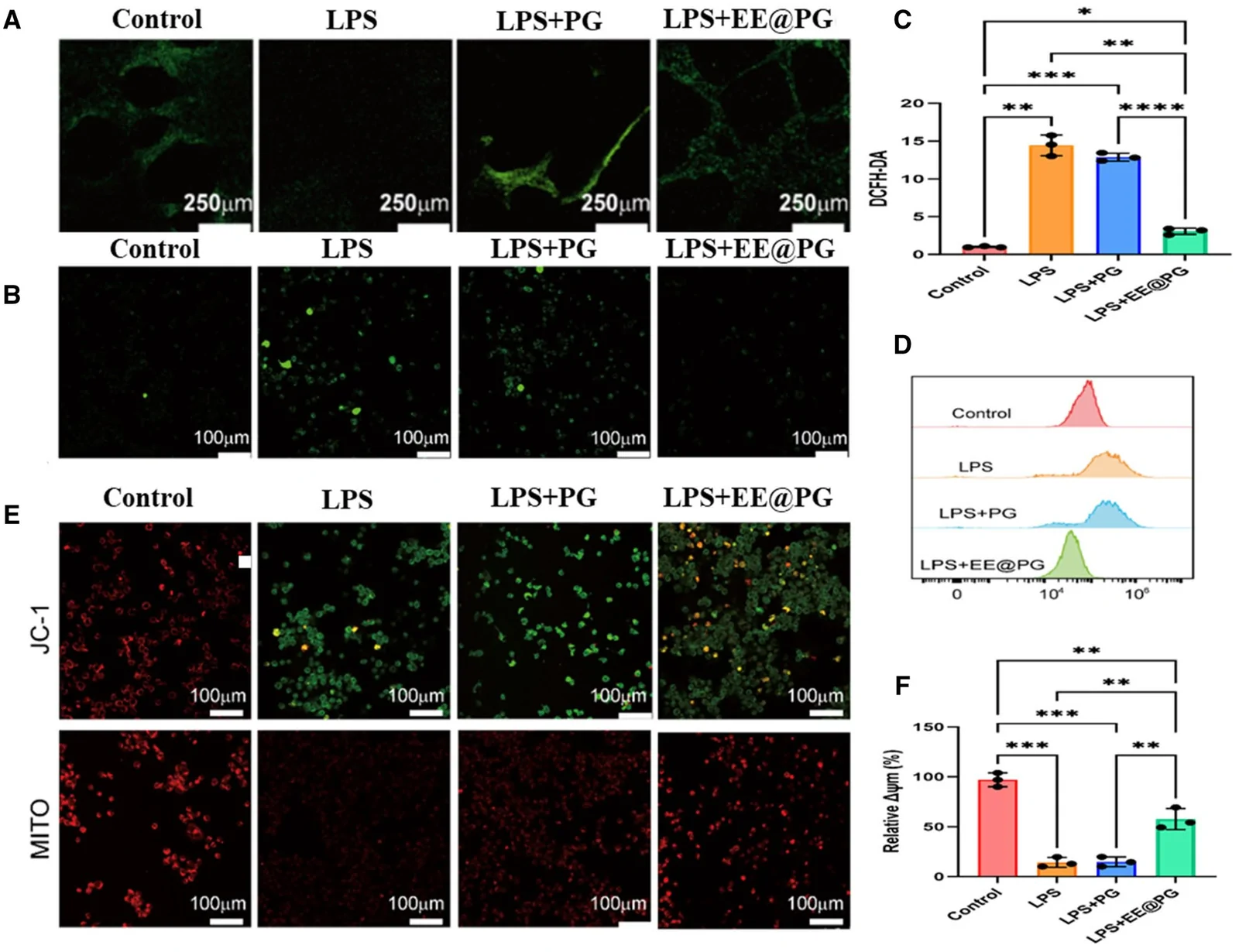
Effects of EE@PG on HUVEC tube formation and on intracellular ROS and mitochondrial membrane potential (ΔΨm) in RAW264.7 macrophages. (**A**) Representative images of HUVEC tube formation after 24 h under the indicated treatments (control, LPS, LPS+PG and LPS+EE@PG). (**B**) Intracellular ROS in RAW264.7 cells visualized by DCFH-DA fluorescence staining and (**C**) corresponding quantitative analysis after 24 h of treatment. (**D**) Flow-cytometric analysis of intracellular ROS levels in RAW264.7 cells after 24 h of treatment. (**E**) Representative fluorescence images of mitochondrial membrane potential (ΔΨm) assessed by JC-1 staining, with MitoTracker as a mitochondrial indicator. (**F**) Quantification of ΔΨm (JC-1 red/green fluorescence ratio). Data are presented as mean ± SD (*n* = 3, technical replicate). *P < 0.05, **P < 0.01, ***P < 0.001, ****P < 0.0001.

### EE@PG reduces intracellular ROS and restores mitochondrial membrane potential in macrophages

Because oxidative stress and mitochondrial dysfunction contribute to persistent inflammation, we next evaluated intracellular ROS and mitochondrial membrane potential (ΔΨm). EE@PG treatment reduced DCFH-DA fluorescence intensity in LPS-stimulated RAW264.7 cells, and this decrease was consistent across fluorescence imaging and flow-cytometric analyses (Figure 4B–D), indicating diminished intracellular ROS burden.

Mitochondrial function was assessed via JC-1 staining. Compared with LPS and LPS+PG groups, EE@PG increased the JC-1 red/green fluorescence ratio, suggesting improved ΔΨm and partial restoration of mitochondrial integrity (Figure 4E and F). These data support a mechanistic link whereby EE@PG mitigates oxidative stress and mitochondrial dysfunction, thereby favoring M2-like macrophage polarization and a pro-repair immune milieu.

### EE@PG accelerates DW closure and improves tissue regeneration *in vivo*

#### EE@PG enhances wound closure and histological repair

The therapeutic efficacy of EE@PG was further evaluated using a splinted full-thickness dorsal wound model in STZ-induced diabetic rats (Figure 5A). Representative wound images and wound-tracing overlays showed faster wound contraction/closure in the EE@PG group than in the PG group over 14 days (Figure 5B and C). Quantification of wound area ratios confirmed accelerated closure with EE@PG at days 3, 7 and 14 (Figure 5D).

**Figure 5:**
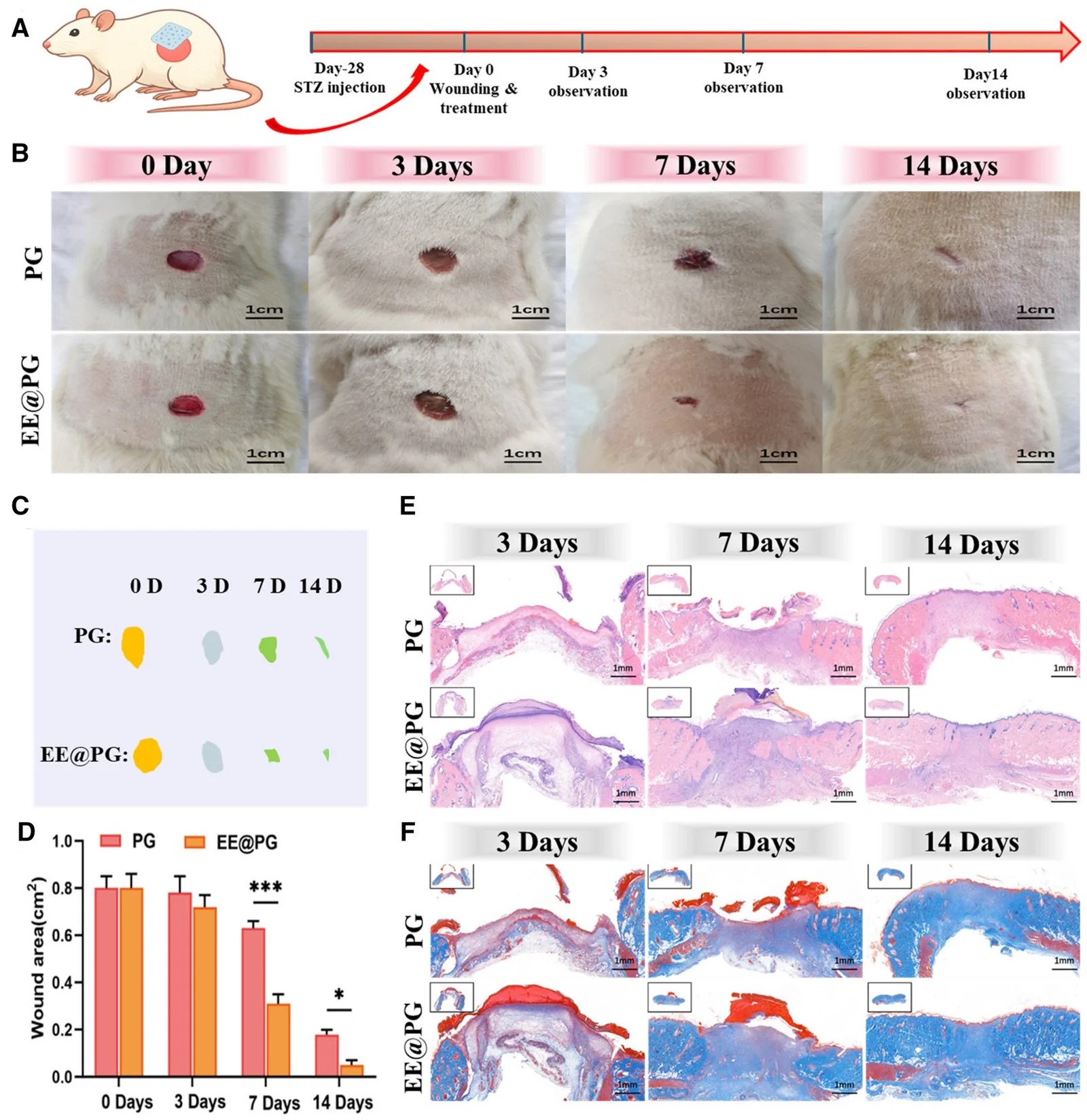
*In vivo* wound healing in STZ-induced diabetic rats treated with PG or EE@PG. (**A**) Schematic timeline of the dorsal full-thickness wound model and sample collection. (**B**) Representative photographs of wounds treated with PG or EE@PG at days 0, 3, 7 and 14 post-surgery. (**C**) Representative wound-tracing overlays showing temporal wound closure; yellow, gray and green outlines indicate wound boundaries at days 0, 3, 7 and 14, respectively. (**D**) Quantification of the wound area at days 0, 3, 7 and 14. (**E**) Representative H&E-stained sections from PG and EE@PG groups at days 3, 7 and 14. (**F**) Representative Masson’s trichrome-stained sections from PG and EE@PG groups at days 3, 7 and 14. Data are presented as mean ± SD (*n* = 3 independent biological replicates). *P < 0.05, ***P < 0.001.

Histological analyses supported improved tissue regeneration. H&E staining revealed thicker granulation tissue formation in the early stage and more advanced re-epithelialization in EE@PG-treated wounds at later stages compared with PG (Figure 5E). Masson’s trichrome staining further demonstrated denser and more organized collagen deposition in EE@PG-treated wounds, consistent with improved matrix remodeling (Figure 5F).

#### EE@PG attenuates inflammation and promotes macrophage M2 polarization in vivo

Inflammation-associated markers were assessed on wound sections at days 7 and 14. Macrophage polarization *in vivo* was examined by immunofluorescence co-staining. EE@PG increased Arg-1^+^ and CD206^+^ signals while reducing iNOS^+^ staining compared with PG, indicating a shift toward an M2-like, pro-repair macrophage phenotype within the wound microenvironment (Figure 6A–D).

**Figure 6:**
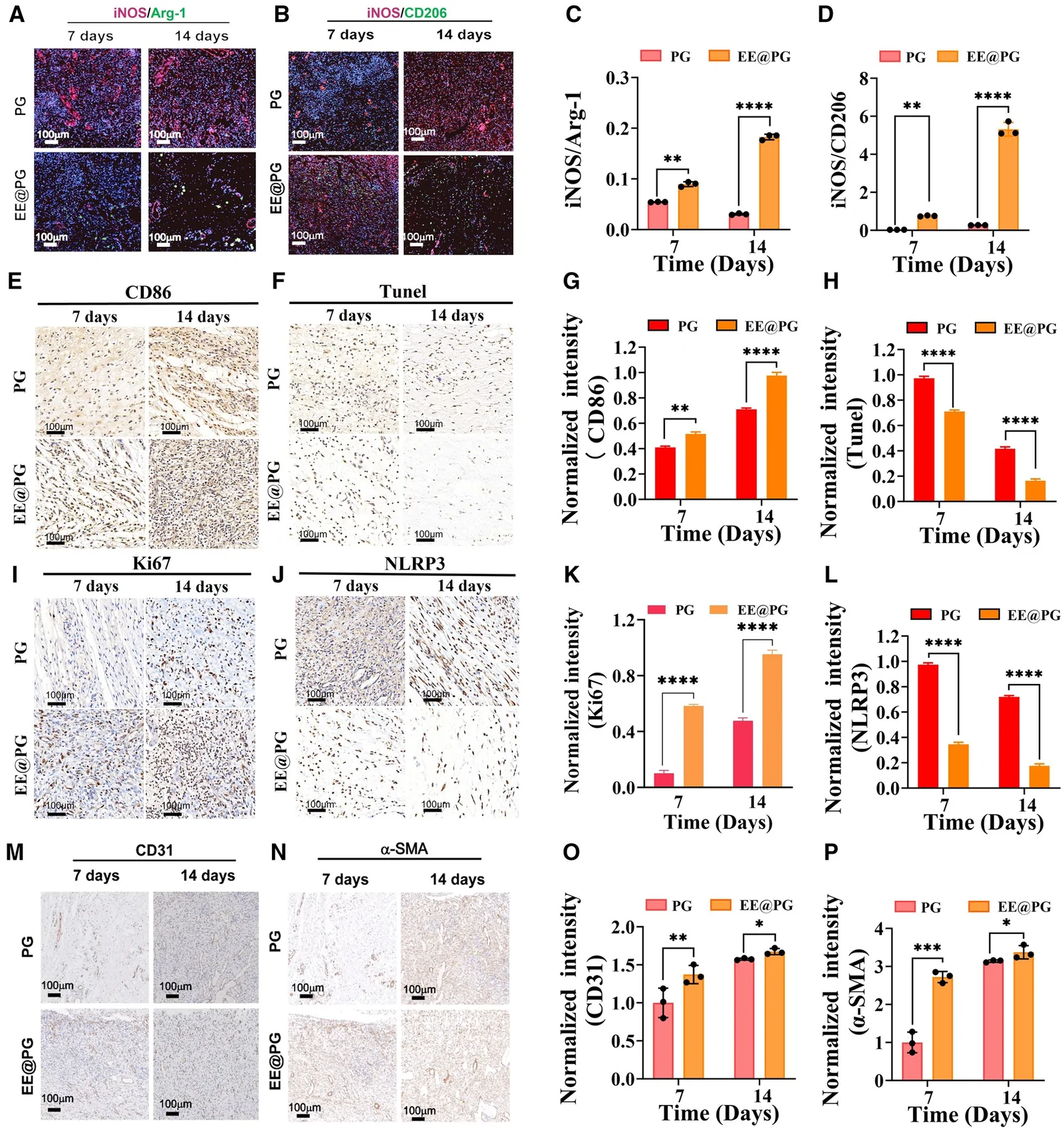
EE@PG promotes macrophage M2 polarization and enhances neovascularization in diabetic wounds. (**A**–**D**) Immunofluorescence images and quantification of macrophage phenotype markers: iNOS/arg-1 and iNOS/CD206 staining in PG and EE@PG groups at days 7 and 14. (**E**–**H**) Representative immunohistochemical images and corresponding quantification of CD86 and TUNEL staining in PG and EE@PG groups at days 7 and 14 post-surgery. (**I**–**L**) Representative immunohistochemical images and corresponding quantification of Ki67 and NLRP3 staining in PG and EE@PG groups at days 7 and 14. (**M**–**P**) Immunofluorescence images and quantification of CD31 and α-SMA staining in PG and EE@PG groups at days 7 and 14, indicating neovascularization and vessel maturation. Data are presented as mean ± SD (*n* = 3 independent biological replicates). *P < 0.05, **P < 0.01, ***P < 0.001, ****P < 0.0001.

Immunohistochemistry showed reduced CD86 staining and fewer TUNEL-positive signals in EE@PG-treated wounds compared with PG, suggesting alleviated inflammatory activation and reduced apoptosis in the wound bed (Figure 6E–H). In addition, EE@PG increased Ki67-positive cells while reducing NLRP3 staining, consistent with enhanced proliferative activity and dampened inflammasome-associated signaling (Figure 6I–L).

#### EE@PG enhances neovascularization and vessel maturation in vivo

Neovascularization and vascular maturation were evaluated by CD31 and α-SMA immunofluorescence staining. EE@PG increased CD31^+^ microvessel density and enhanced α-SMA^+^ perivascular coverage at days 7 and 14 relative to PG (Figure 6M–P), indicating both increased angiogenesis and improved vessel maturation. These *in vivo* findings align with the *in vitro* tube formation results (Figure 4A), supporting an EE@PG-driven pro-angiogenic effect.

### EE@PG regulates ECM remodeling genes and upregulates VEGF/fibronectin signaling

To further probe molecular programs associated with tissue repair, qRT-PCR was performed on wound tissues. EE@PG increased the expression of ECM remodeling-related genes, including *Collagen I* and *MMP2*, compared with PG across days 3, 7 and 14 (Figure 7A and B).

**Figure 7:**
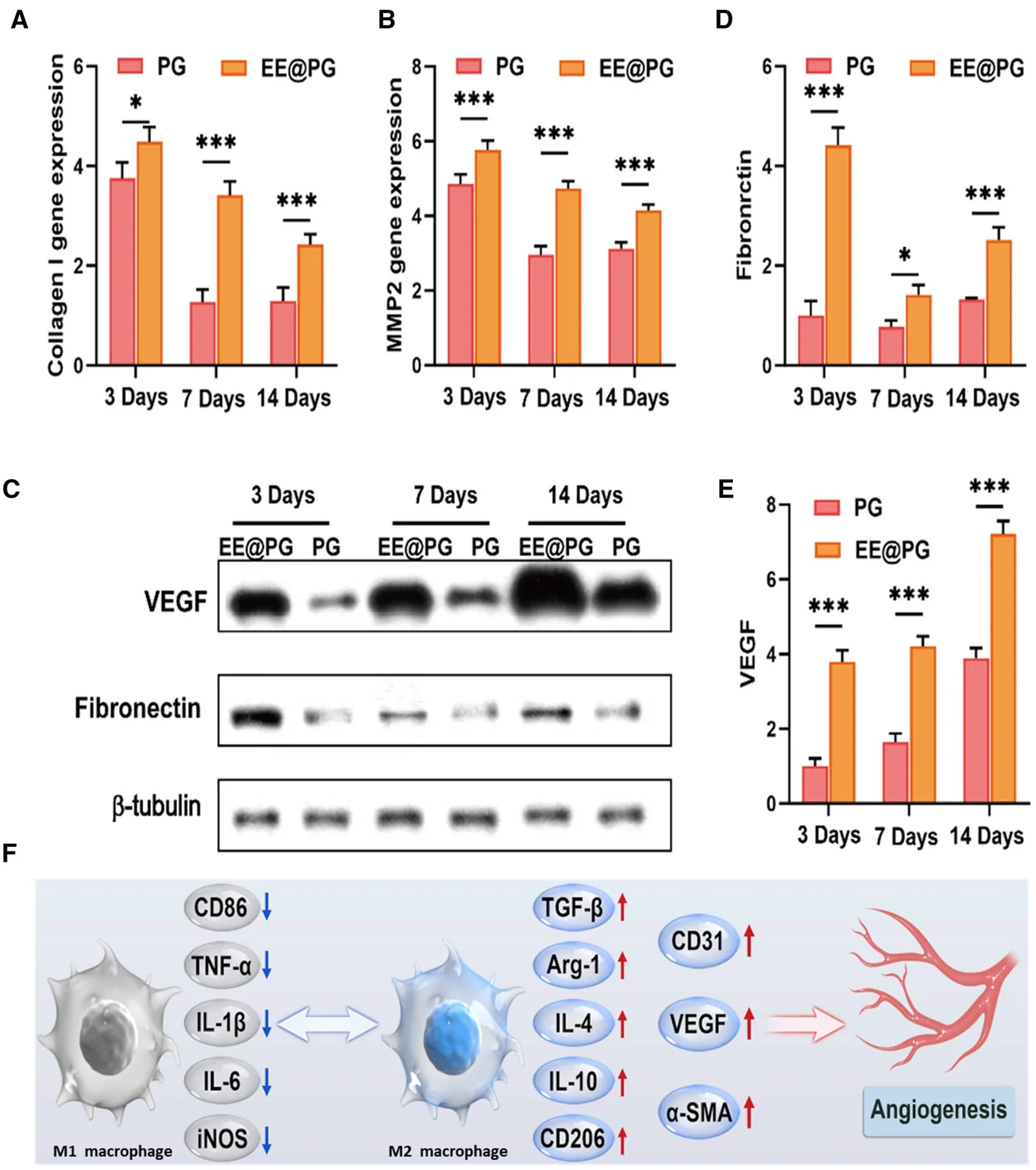
Gene expression and VEGF/fibronectin signaling associated with EE@PG-mediated wound repair. (**A** and **B**) qRT-PCR analysis of extracellular matrix remodeling-related genes, including (**A**) *Collagen I* and (**B**) *MMP2*, in wound tissues from PG and EE@PG groups at days 3, 7 and 14 post-surgery. (**C**) Representative Western blots of VEGF, fibronectin and β-tubulin in PG and EE@PG groups at days 3, 7 and 14. (**D** and **E**) Densitometric quantification of (**D**) fibronectin and (**E**) VEGF normalized to β-tubulin. (**F**) Proposed mechanism by which EE@PG attenuates pro-inflammatory M1-associated markers (CD86, TNF-α, IL-1β, IL-6 iNOS) while enhancing M2-associated repair markers (TGF-β, arg-1, IL-4, IL-10, CD206), thereby promoting angiogenesis and vascular maturation (CD31, VEGF, α-SMA). Data are presented as mean ± SD (*n* = 3 independent biological replicates). *P < 0.05, ***P < 0.001.

Western blot analyses further showed higher VEGF and fibronectin protein levels in the EE@PG group than in the PG group, with β-tubulin serving as a loading control (Figure 7C–E). Together, these results support an EE@PG-associated shift toward a pro-matrix, pro-angiogenic wound microenvironment.

As summarized in Figure 7F, EE@PG treatment was associated with a coordinated shift toward a pro-repair wound microenvironment, characterized by suppression of M1-associated inflammatory markers (e.g., CD86, TNF-α, IL-1β, IL-6 and iNOS) and enhancement of M2-associated repair markers (e.g., TGF-β, Arg-1, IL-4, IL-10 and CD206). This immunomodulatory profile coincided with increased expression of angiogenesis- and vascular maturation-related markers, including CD31, VEGF, and α-SMA, consistent with the immunostaining and protein analyses in Figures 6 and 7.

### RNA-seq reveals transcriptomic reprogramming toward immune resolution and tissue repair

Bulk RNA sequencing was conducted to compare transcriptional landscapes among control, DW and EE@PG-treated tissues. Hierarchical clustering demonstrated distinct expression patterns across the three groups (Figure 8A). The Venn analysis indicated both shared and group-specific differentially expressed genes (DEGs) (Figure 8B).

**Figure 8:**
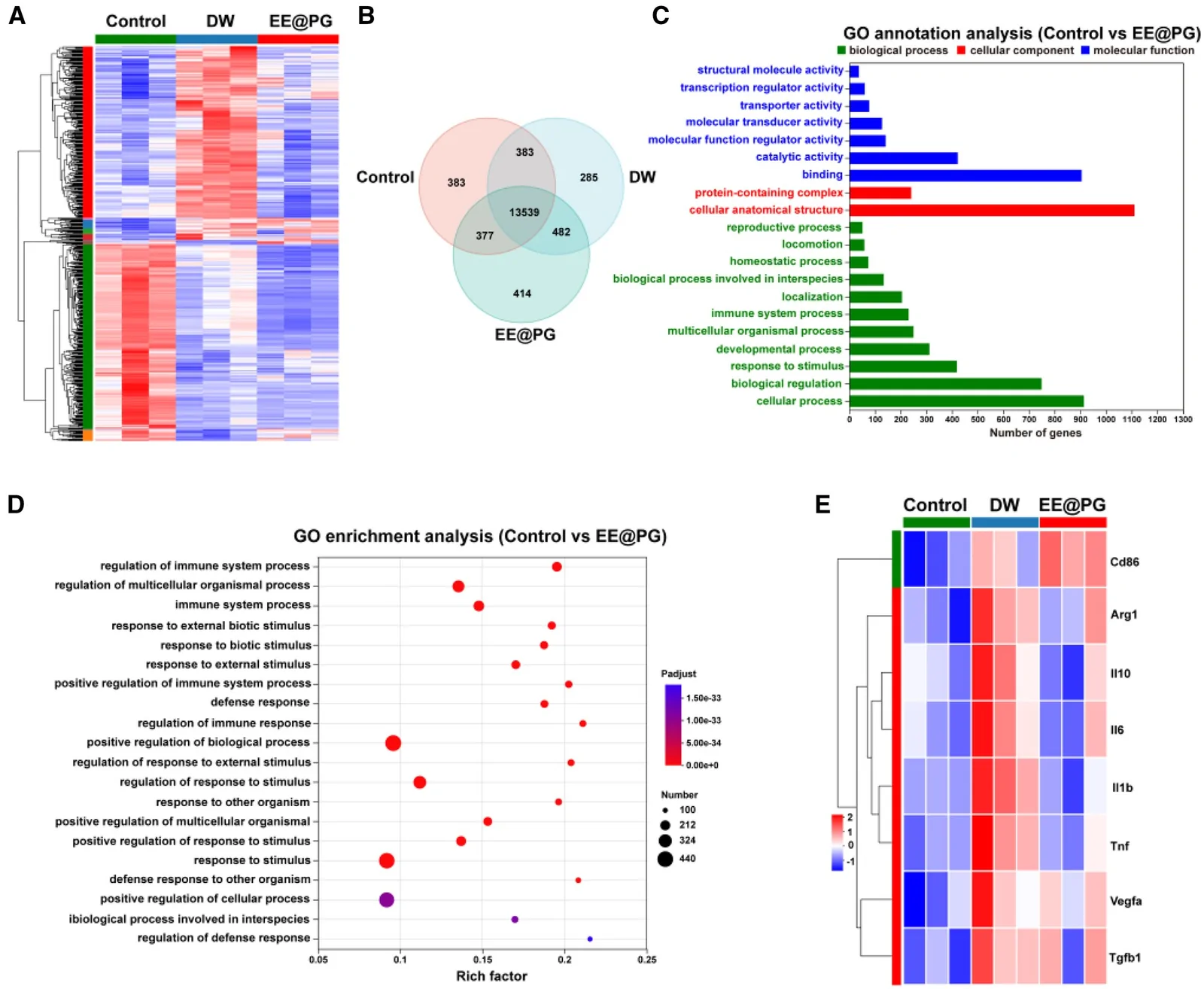
Transcriptomic similarities and differences among control, DW and EE@PG tissues. (**A**) Hierarchical clustering heatmap of differentially expressed genes (DEGs) among control, DW and EE@PG tissues. (**B**) Venn diagram showing the overlap of DEGs among control, DW and EE@PG groups. (**C**) GO enrichment clustering analysis comparing the control and EE@PG groups. (**D**) Bubble plot of significantly enriched GO terms between control and EE@PG groups. (**E**) Heatmap of immune-related gene expression comparing control and EE@PG tissues.

GO enrichment analyses comparing Control and EE@PG highlighted biological processes associated with immune regulation and responses to external stimuli (Figure 8C and D). Further GO enrichment analysis with directional annotation showed that, in the comparison between the EE@PG and Control groups, the up-regulated DEGs were mainly enriched in biological processes related to tissue repair and adaptive regulation, whereas the down-regulated DEGs were primarily enriched in immune- and defense-response-related processes (Supplementary Figure S9). A focused heatmap of immune-related genes further suggested that EE@PG shifted the wound transcriptome toward reduced inflammatory activation and enhanced repair-associated programs (Figure 8E), consistent with the immunostaining and functional outcomes described above.

### Hemocompatibility and in vivo biosafety

Systemic safety was assessed to support translational feasibility. H&E staining of major organs (heart, liver and spleen) at days 7 and 14 showed no apparent histopathological abnormalities in either PG- or EE@PG-treated rats (Figure 9A). Hemolysis testing indicated low hemolytic activity across tested conditions (Figure 9B). Routine blood parameters at days 7 and 14 showed no marked hematological abnormalities, supporting acceptable hemocompatibility and systemic safety within the study period (Figure 9C). Consistently, body weight changes did not differ notably between groups during the 14-day observation window (Supplementary Figure S10).

**Figure 9:**
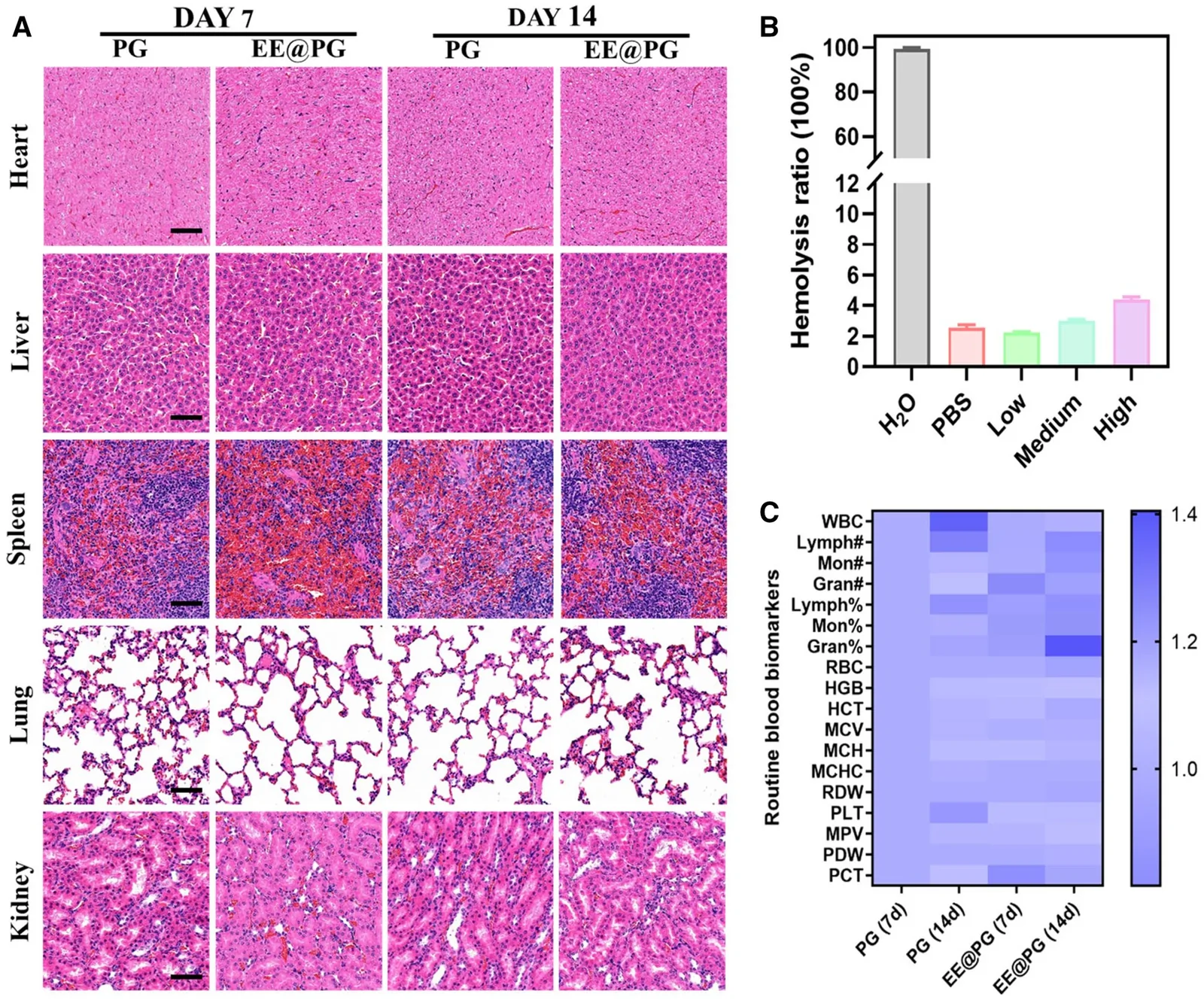
Hemocompatibility and *in vivo* biosafety of PG and EE@PG nanofibrous mats. (**A**) Representative H&E-stained sections of major organs (heart, liver, spleen, lung and kidney) from PG- and EE@PG-treated rats at days 7 and 14 post-surgery, showing no apparent histopathological abnormalities. Scale bar = 50 μm. (**B**) Hemolysis assay results. (**C**) Routine hematological parameters measured at days 7 and 14 in PG and EE@PG groups, indicating good hemocompatibility and systemic safety.

## Discussion

### Principal findings

This study demonstrates that an EPC-Exos-functionalized, aligned PLGA/gelatin nanofibrous scaffold (EE@PG) accelerates repair of full-thickness diabetic skin wounds through coordinated immunomodulation and angiogenic activation. *In vitro*, EE@PG reduced oxidative stress in macrophages, promoted a shift of RAW264.7 cells toward an M2-like phenotype (increased CD206 and Arg-1, decreased iNOS and CCR7), and rebalanced inflammatory cytokines by elevating IL-10 and IL-4 while reducing TNF-α and IL-6. EE@PG also improved endothelial angiogenic function under inflammatory challenge, as evidenced by rescue of HUVEC tube formation. *In vivo*, EE@PG reduced residual wound area, promoted re-epithelialization and collagen remodeling, and increased CD31-positive vessel density with enhanced α-SMA-positive perivascular coverage, indicating improved vascular maturation. Tissue-level qRT-PCR and Western blot analyses supported these outcomes by showing higher Collagen I and MMP2 expression and increased VEGF and fibronectin levels, while bulk RNA sequencing indicated a transcriptional shift toward immune resolution and pro-repair programs. Collectively, these findings support a mechanism in which local, sustained delivery of EPC-Exos from an aligned nanofibrous matrix attenuates redox-associated inflammation, biases macrophage polarization toward a reparative state, and secondarily enhances endothelial activity, leading to accelerated wound closure. Notably, the *in vitro* macrophage data support an immunomodulatory role of EE@PG in promoting a pro-repair phenotype, whereas the increased CD31 and α-SMA expression observed *in vivo*, together with the enhanced tube formation *in vitro*, suggest that this pro-repair microenvironment is associated with improved angiogenesis and vascular maturation, although a direct causal relationship has not yet been definitively established.

### Context within the literature

Exosomes and other EVs are increasingly recognized as paracrine effectors that can modulate chronic wound microenvironments by tuning macrophage plasticity and promoting neovascularization [38]. Multiple delivery formats, such as hydrogels, films, and fibrous scaffolds, have been explored for EV-based wound repair; however, simultaneous control over dose, spatial presentation, and release kinetics while preserving EV bioactivity remains a key translational challenge. This need for rigor in EV identity, characterization, and functional claims is emphasized by the most recent EV reporting standards [39].

The present work contributes three advances. First, the aligned PLGA/gelatin architecture provides a high surface area, ECM–mimetic interface that supports cell adhesion and local retention of bioactive cues, consistent with the broader utility of electrospun nanofiber dressings in DW repair [40]. Second, sustained local presentation from EE@PG helps address rapid clearance and short-lived exposure associated with freely administered EVs, thereby maintaining bioactive signaling across the early inflammatory and subsequent proliferative phases. Third, a coherent multi-scale linkage is established: reduction of ROS and improved mitochondrial function align with macrophage polarization toward a reparative phenotype, which is accompanied by improved endothelial angiogenic behavior *in vitro* and enhanced neovascularization and vessel maturation *in vivo*.

### Mechanistic considerations

The data are consistent with a model in which EPC-Exos interrupt redox-inflammatory amplification in DWs. Reduced DCFH-DA signal and improved JC-1 red/green ratios indicate that EE@PG mitigates mitochondrial depolarization, a characteristic feature of hyperglycemia-associated cellular stress. The accompanying decreases in NLRP3 and CD86 signals suggest a reduction in pro-inflammatory activation that is compatible with a shift toward an IL-10 and TGF-β-skewed reparative milieu. In parallel, the endothelial compartment exhibits enhanced pro-angiogenic remodeling, supported by increased VEGF and fibronectin protein expression and improved tube formation under inflammatory stress. These observations are consistent with prior evidence that EV-mediated signaling can promote angiogenesis and modulate macrophage phenotype in DW settings [41].

While this work did not profile EPC-Exos cargo, previous studies indicate that EV-associated proteins and regulatory RNAs can converge on pathways linked to redox homeostasis and angiogenic signaling, including PI3K/Akt/eNOS and ECM–integrin interactions [42, 43]. Future targeted profiling of EPC-Exos cargo, together with pathway-level perturbation, would strengthen causal attribution and enable rational potency assays aligned with quality-by-design principles.

### Rationale for aligned PLGA/gelatin nanofibers

The PLGA/gelatin composite combines PLGA’s mechanical robustness and tunable degradation with gelatin’s hydrophilicity and cell-recognition motifs, thereby improving wettability and supporting interactions relevant to both immune and endothelial compartments [44]. Fiber alignment can bias cell morphology and migration and may facilitate directional endothelial organization, providing a physical cue that complements the biochemical cues delivered by EPC-Exos. Compared with isotropic hydrogels, anisotropic fibrous networks also offer predictable diffusion pathways and interfiber voids that can support sustained release and local retention, while partially shielding EVs from enzymatic degradation in the wound milieu. The broader literature supports electrospun nanofiber dressings as an adaptable platform for DW management, particularly when combined with pro-regenerative bioactive factors [40].

### Translational relevance and manufacturability

From a translational perspective, both PLGA and gelatin have extensive precedents in biomedical devices and wound care. The scaffold architecture is compatible with scalable electrospinning and roll-to-roll collection, and exosome functionalization can be performed by aqueous post-loading to minimize disruption of EV integrity. For clinical advancement, a GMP-oriented quality plan is recommended, including EV identity and purity assessments aligned with current standards (MISEV2018 and MISEV2023), such as NTA, TEM, immunoblotting for positive markers (CD9, CD63, CD81) and a negative marker, endotoxin testing by LAL, and sterility testing. In addition, fit-for-purpose potency assays (e.g., a macrophage polarization index or an endothelial tube formation score), dose normalization (particles per area and per dressing), and validated cumulative release profiles would help define critical quality attributes. Early consideration of sterilization compatibility and stability (including post-sterilization bioactivity) and ISO 10993-aligned biocompatibility will further support regulatory progression, which is likely to follow a combination-product pathway.

### Limitations

Several limitations warrant consideration. (i) Although the study uses an STZ-induced diabetic rat model, species and model descriptors must be consistently presented across Methods, Results, and figure legends, together with explicit randomization and blinding statements following ARRIVE 2.0. (ii) While group differences are robust, reporting effect sizes, confidence intervals, and exact *P-*value for primary and secondary endpoints, alongside multiplicity control where appropriate, would strengthen statistical rigor. (iii) A direct exposure–response analysis linking cumulative exosome release to angiogenesis or closure metrics was not performed; such analyses would reinforce claims regarding sustained delivery. (iv) EV heterogeneity was not characterized at the cargo level, and donor or batch variability may affect potency. (v) The *in vivo* window ends at day 14; longer-term outcomes such as scar quality, tensile strength, remodeling, and biodistribution/toxicology remain to be determined. (vi) Additional controls, such as heat-inactivated EVs, EV-depleted conditioned medium, and a dose-matched free-EV dressing arm, would further disentangle scaffold versus EV contributions. (vii) Although the present study suggests that EE@PG exerts coordinated pro-repair effects through modulation of oxidative stress, mitochondrial function, macrophage polarization, and angiogenesis, the absence of key comparator groups, including free EVs, EV-depleted conditioned medium, and inactivated EVs, means that the current evidence remains primarily associative and does not yet allow strict separation of scaffold effects from exosome-specific bioactivity. (viii) Sterilization methods should be clearly documented and clinically relevant, and residuals and post-sterilization EV bioactivity should be evaluated as part of translational readiness. (ix) In addition, the *in vivo* DW model did not include an untreated blank control group, which limits direct assessment of the baseline spontaneous healing response and reduces the ability to fully distinguish natural wound closure from scaffold-mediated therapeutic effects. Future studies should incorporate an untreated DW control to provide a more complete benchmark for evaluating treatment efficacy.

### Future work

Future studies should (i) profile EPC-Exos cargo (miRNA and protein) and link specific pathways to the observed phenotypes; (ii) use single-cell or spatial transcriptomics to map macrophage and endothelial states within the wound microenvironment; (iii) evaluate large-animal diabetic models and infected wound settings to better approximate clinical complexity; (iv) optimize dressing format parameters (conformability, moisture handling, wear time) and practical clinical workflows (including dressing change intervals); (v) build mechanistic PK/PD models connecting release kinetics to potency readouts and healing trajectories; and (vi) advance GMP manufacturing, including lot-release potency assays and stability-indicating tests under accelerated and real-time conditions.

## Conclusions

In conclusion, we developed an exosome-enabled, aligned PLGA/gelatin nanofibrous dressing (EE@PG) designed to address the key pathological barriers of DW healing. By integrating an ECM–mimetic fibrous architecture with localized, sustained delivery of EPC-derived exosomes, EE@PG provided both structural support and prolonged bioactive signaling at the wound site. *In vitro*, EE@PG showed good cytocompatibility, reduced oxidative stress, restored mitochondrial membrane potential, promoted macrophage polarization toward a pro-repair M2 phenotype and improved endothelial angiogenic function under inflammatory conditions. In a splinted full-thickness STZ-induced diabetic rat model, EE@PG significantly accelerated wound closure, enhanced re-epithelialization and collagen remodeling, increased neovascularization and vessel maturation, and attenuated inflammatory responses while demonstrating favorable hemocompatibility and systemic biosafety. Transcriptomic analyses further supported a global shift toward immune resolution and tissue repair programs following EE@PG treatment. Together, these findings highlight a generalizable design principle for drug-delivering, exosome-functionalized nanofibrous dressings that coordinately modulate immunity and angiogenesis, offering a promising and translatable therapeutic strategy for chronic DWs and other hard-to-heal tissue defects.

## Supplementary Material

rbag116_Supplementary_Data

## References

[1] Chen G , Zhu X, Yang L, He T, Huang B, Ou X, Chen L, Yan T, Zhang S, Lyu G, Xu Y, Dai W, Xia H, Yu D. A strategy for diabetic wound healing: electrostatic adsorption of structural stability drugs with antibacterial, anti-inflammatory, and angiogenic effects. Adv Funct Mater 2026;36:e25109.

[2] Chen L , Cao P, Zhao P, Xu Y, Lv G, Yu D. Photodynamic-controllable microneedle composite with antibacterial, antioxidant, and angiogenic effects to expedite infected diabetic wound healing. Mater Des 2024;241:112971.

[3] Peña OA , Martin P. Cellular and molecular mechanisms of skin wound healing. Nat Rev Mol Cell Biol 2024;25:599–616. 10.1038/s41580-024-00715-1 38528155

[4] Huang K , Mi B, Xiong Y, Fu Z, Zhou W, Liu W, Liu G, Dai G. Angiogenesis during diabetic wound repair: from mechanism to therapy opportunity. Burns Trauma 2025; 13: tkae052. 10.1093/burnst/tkae052 PMC11802347 39927093

[5] Han X , Saengow C, Ju L, Ren W, Ewoldt RH, Irudayaraj J. Exosome-coated oxygen nanobubble-laden hydrogel augments intracellular delivery of exosomes for enhanced wound healing. Nat Commun 2024;15:3435. 10.1038/s41467-024-47696-5 PMC11039765 38653959

[6] Lai CM , Chen WJ, Qin Y, Xu D, Lai YK, He SH. Innovative hydrogel design: tailoring immunomodulation for optimal chronic wound recovery. Adv Sci (Weinh) 2025;12:e2412360. 10.1002/advs.202412360 PMC11727140 39575827

[7] Nayer B , Tan JL, Alshoubaki YK, Lu YZ, Legrand JMD, Lau S, Hu N, Park AJ, Wang XN, Amann-Zalcenstein D, Hickey PF, Wilson T, Kuhn GA, Müller R, Vasanthakumar A, Akira S, Martino MM. Local administration of regulatory T cells promotes tissue healing. Nat Commun 2024;15:7863. 10.1038/s41467-024-51353-2 PMC11383969 39251592

[8] Wang Y , Vizely K, Li CY, Shen K, Shakeri A, Khosravi R, Smith JR, Alteza E, Zhao Y, Radisic M. Biomaterials for immunomodulation in wound healing. Regen Biomater 2024;11:rbae032. 10.1093/rb/rbae032 PMC11110865 38779347

[9] Zhu D , Hu Y, Kong X, Luo Y, Zhang Y, Wu Y, Tan J, Chen J, Xu T, Zhu L. Enhanced burn wound healing by controlled-release 3D ADMSC-derived exosome-loaded hyaluronan hydrogel. Regen Biomater 2024;11:rbae035. 10.1093/rb/rbae035 PMC11018541 38628545

[10] Bhattacharjee S , Gao J, Lu YW, Eisa-Beygi S, Wu H, Li K, Birsner AE, Wong S, Song Y, Shyy JY, Cowan DB, Huang W, Wei W, Aikawa M, Shi J, Chen H. Endothelial FOXM1 and Dab2 promote diabetic wound healing. JCI Insight 2025;10:e186504. 10.1172/jci.insight.186504 PMC11790024 39846251

[11] Yan L , Wang J, Cai X, Liou YC, Shen HM, Hao J, Huang C, Luo G, He W. Macrophage plasticity: signaling pathways, tissue repair, and regeneration. MedComm (2020) 2024;5:e658. 10.1002/mco2.658 PMC11292402 39092292

[12] Xia H , Zhang Y, Xin H, Yan D, Li G, Chen R. Metal–phenolic network-based polydopamine@Cu within a polyvinyl alcohol hydrogel film for improved infected wound healing through antibacterial and pro-angiogenesis activity. Mater Des 2022;221:110904.

[13] Lauterbach AL , Wallace RP, Alpar AT, Refvik KC, Reda JW, Ishihara A, Beckman TN, Slezak AJ, Mizukami Y, Mansurov A, Gomes S, Ishihara J, Hubbell JA. Topically-applied collagen-binding serum albumin-fused interleukin-4 modulates wound microenvironment in non-healing wounds. NPJ Regen Med 2023;8:49. 10.1038/s41536-023-00326-y PMC10495343 37696884

[14] McDermott K , Fang M, Boulton AJM, Selvin E, Hicks CW. Etiology, epidemiology, and disparities in the burden of diabetic foot ulcers. Diabetes Care 2023;46:209–21. 10.2337/dci22-0043 PMC9797649 36548709

[15] Lu W , Du X, Zou S, Fang Q, Wu M, Li H, Shi B. IFN-γ enhances the therapeutic efficacy of MSCs-derived exosome via miR-126-3p in diabetic wound healing by targeting SPRED1. J Diabetes 2024;16:e13465. 10.1111/1753-0407.13465 PMC10809290 37646268

[16] Li P , Hong G, Zhan W, Deng M, Tu C, Wei J, Lin H. Endothelial progenitor cell derived exosomes mediated miR-182-5p delivery accelerate diabetic wound healing via down-regulating PPARG. Int J Med Sci 2023;20:468–81. 10.7150/ijms.78790 PMC10087624 37057206

[17] Zhang H , Lu C, Wu L, Li J, Huang M, Tao X, Wu Y, Jia B. Exosomes derived from endothelial progenitor cells ameliorate LPS-induced brain microvascular endothelial cells injury by delivering miR-126a-5p. Sci Rep 2024;14:18469. 10.1038/s41598-024-69163-3 PMC11316067 39122748

[18] Liang G , Zeng M, Wang Q, Wang W, Liu J, Gao M, He Z. Exosomal miR-218 secreted from endothelial progenitor cells mitigates acute lung injury in sepsis mice by inhibiting HMGA1 in alveolar macrophages. Stem Cell Res Ther 2025;16:431. 10.1186/s13287-025-04558-1 PMC12330018 40770661

[19] Kwak G , Cheng J, Kim H, Song S, Lee SJ, Yang Y, Jeong JH, Lee JE, Messersmith PB, Kim SH. Sustained Exosome-Guided macrophage polarization using hydrolytically degradable PEG hydrogels for cutaneous wound healing: identification of key proteins and MiRNAs, and sustained release formulation. Small 2022; 18: e2200060. 10.1002/smll.202200060 35229462

[20] Song Y , You Y, Xu X, Lu J, Huang X, Zhang J, Zhu L, Hu J, Wu X, Xu X, Tan W, Du Y. Adipose-derived mesenchymal stem cell-derived exosomes biopotentiated extracellular matrix hydrogels accelerate diabetic wound healing and skin regeneration. Adv Sci (Weinh) 2023;10:e2304023. 10.1002/advs.202304023 PMC10602544 37712174

[21] Li X , Wang Y, Shi L, Li B, Li J, Wei Z, Lv H, Wu L, Zhang H, Yang B, Xu X, Jiang J. Magnetic targeting enhances the cutaneous wound healing effects of human mesenchymal stem cell-derived iron oxide exosomes. J Nanobiotechnology 2020;18:113. 10.1186/s12951-020-00670-x PMC7429707 32799868

[22] Wiegand C , Abel M, Hipler UC, Elsner P. Effect of non-adhering dressings on promotion of fibroblast proliferation and wound healing in vitro. Sci Rep 2019;9:4320. 10.1038/s41598-019-40921-y PMC6416289 30867534

[23] Diao Z , Li L, Zhou H, Yang L. Tannic acid and silicate-functionalized polyvinyl alcohol-hyaluronic acid hydrogel for infected diabetic wound healing. Regen Biomater 2024;11:rbae053. 10.1093/rb/rbae053 PMC11176089 38883183

[24] Li M , Xia W, Khoong YM, Huang L, Huang X, Liang H, Zhao Y, Mao J, Yu H, Zan T. Smart and versatile biomaterials for cutaneous wound healing. Biomater Res 2023;27:87. 10.1186/s40824-023-00426-2 PMC10504797 37717028

[25] Zhao X , Dai W, Liu C, An M, Li S, Guo L, Fan Y, Zhang X. Gelatin/hyaluronic acid-based in situ forming hydrogel promotes wound regeneration by the synergy of ROS-scavenging and pro-healing activity. Regen Biomater 2025;12:rbaf052. 10.1093/rb/rbaf052 PMC12286701 40703206

[26] Cao Y , Wang L, Zhang X, Lu Y, Wei Y, Liang Z, Hu Y, Huang D. Double-crosslinked PNIPAM-based hydrogel dressings with adjustable adhesion and contractility. Regen Biomater 2023;10:rbad081. 10.1093/rb/rbad081 PMC10570987 37840848

[27] Gentile P , Chiono V, Carmagnola I, Hatton PV. An overview of poly(lactic-co-glycolic) acid (PLGA)-based biomaterials for bone tissue engineering. Int J Mol Sci 2014;15:3640–59. 10.3390/ijms15033640 PMC3975359 24590126

[28] Yan B , Hua Y, Wang J, Shao T, Wang S, Gao X, Gao J. Surface modification progress for PLGA-Based cell scaffolds. Polymers (Basel) 2024;16:165. 10.3390/polym16010165 PMC10780542 38201830

[29] Dai X , Nie W, Shen H, Machens HG, Böker K, Taheri S, Lehmann W, Shen Y, Schilling AF. Electrospinning based biomaterials for biomimetic fabrication, bioactive protein delivery and wound regenerative repair. Regen Biomater 2025;12:rbae139. 10.1093/rb/rbae139 PMC11723536 39803356

[30] Xu Y , Duan L, Li Y, She Y, Zhu J, Zhou G, Jiang G, Yang Y. Nanofibrillar decellularized wharton’s jelly matrix for segmental tracheal repair. Adv Funct Materials 2020;30:1910067.

[31] Lin Z , Zhong R, Xu Y, Wu Y, Ru C. Shell-core structured nanofibers mediate staged anti-inflammatory and pro-neurogenic activities to repair peripheral nerve. Mater Res Express 2024;11:085006.

[32] Ni B , Ma H, Zhang S, Chengliu H, Xu Y, Dai W, Min P, Li L. Shell-core-structured electrospinning film with sequential anti-inflammatory and pro-neurogenic effects for peripheral nerve repairment. Nanotechnol Rev 2024;13:563–77.

[33] Zhou T , Chen Y, Fu L, Wang S, Ding H, Bai Q, Guan J, Mao Y. In situ MgO nanoparticle-doped janus electrospun dressing against bacterial invasion and immune imbalance for irregular wound healing. Regen Biomater 2024;11:rbae107. 10.1093/rb/rbae107 PMC11379472 39246578

[34] Gainza G , Villullas S, Pedraz JL, Hernandez RM, Igartua M. Advances in drug delivery systems (DDSs) to release growth factors for wound healing and skin regeneration. Nanomedicine 2015;11:1551–73. 10.1016/j.nano.2015.03.002 25804415

[35] Gao E , Wang Z, Zhao S, Zhu X, Xu S, Xu Y, Wang Y, Xu N, Tao B. Core-shell structured nanomembrane with sequential immune and vascular isolation effects followed by chondrogenic induction to promote stable stem cell-based subcutaneous cartilage regeneration in large animals. Chem Eng J 2024;498:154878.

[36] He W , Li C, Zhao S, Li Z, Wu J, Li J, Zhou H, Yang Y, Xu Y, Xia H. Integrating coaxial electrospinning and 3D printing technologies for the development of biphasic porous scaffolds enabling spatiotemporal control in tumor ablation and osteochondral regeneration. Bioact Mater 2024;34:338–53. 10.1016/j.bioactmat.2023.12.020 PMC10809007 38274295

[37] Huang L , Guo Z, Yang X, Zhang Y, Liang Y, Chen X, Qiu X, Chen X. Advancements in GelMA bioactive hydrogels: strategies for infection control and bone tissue regeneration. Theranostics 2025;15:460–93. 10.7150/thno.103725 PMC11671377 39744697

[38] Lyu L , Cai Y, Zhang G, Jing Z, Liang J, Zhang R, Dang X, Zhang C. Exosomes derived from M2 macrophages induce angiogenesis to promote wound healing. Front Mol Biosci 2022;9:1008802. 10.3389/fmolb.2022.1008802 PMC9592913 36304927

[39] Welsh JA , Goberdhan DCI, O’Driscoll L, Buzas EI, Blenkiron C, Bussolati B, Cai H, Di Vizio D, Driedonks TAP, Erdbrügger U, Falcon-Perez JM, Fu Q-L, Hill AF, Lenassi M, Lim SK, Mahoney MG, Mohanty S, Möller A, Nieuwland R, Ochiya T, Sahoo S, Torrecilhas AC, Zheng L, Zijlstra A, Abuelreich S, Bagabas R, Bergese P, Bridges EM, Brucale M, Burger D, Carney RP, Cocucci E, Crescitelli R, Hanser E, Harris AL, Haughey NJ, Hendrix A, Ivanov AR, Jovanovic-Talisman T, Kruh-Garcia NA, Ku’ulei-Lyn Faustino V, Kyburz D, Lässer C, Lennon KM, Lötvall J, Maddox AL, Martens-Uzunova ES, Mizenko RR, Newman LA, Ridolfi A, Rohde E, Rojalin T, Rowland A, Saftics A, Sandau US, Saugstad JA, Shekari F, Swift S, Ter-Ovanesyan D, Tosar JP, Useckaite Z, Valle F, Varga Z, van der Pol E, van Herwijnen MJC, Wauben MHM, Wehman AM, Williams S, Zendrini A, Zimmerman AJ, Consortium M, Théry C, Witwer KW; MISEV Consortium. Minimal information for studies of extracellular vesicles (MISEV2023): from basic to advanced approaches. J Extracell Vesicles 2024;13:e12404. 10.1002/jev2.12404 PMC10850029 38326288

[40] Jang EJ , Patel R, Patel M. Electrospinning nanofibers as a dressing to treat diabetic wounds. Pharmaceutics 2023;15:1144. 10.3390/pharmaceutics15041144 PMC10142830 37111630

[41] Cheng P , Xie X, Hu L, Zhou W, Mi B, Xiong Y, Xue H, Zhang K, Zhang Y, Hu Y, Chen L, Zha K, Lv B, Lin Z, Lin C, Dai G, Hu Y, Yu T, Hu H, Liu G, Zhang Y. Hypoxia endothelial cells-derived exosomes facilitate diabetic wound healing through improving endothelial cell function and promoting M2 macrophages polarization. Bioact Mater 2024;33:157–73. 10.1016/j.bioactmat.2023.10.020 PMC10681882 38034500

[42] Li Z , Bai Y, Wu H, Feng Y, Wang X, Zhao C, Wang X. PTEN/PI3K/AKT pathway activation with hypoxia-induced human umbilical vein endothelial cell exosome for angiogenesis-based diabetic skin reconstruction. Mater Today Bio 2025;32:101651. 10.1016/j.mtbio.2025.101651 PMC11964554 40177380

[43] Chowdhury S , Kumar A, Patel P, Kurmi BD, Jain S, Kumar B, Vaidya A. Stem cell-derived exosomes for diabetic wound healing: mechanisms, nano-delivery systems, and translational perspectives. JNT 2026;7:1.

[44] Guo Z , Zhu X, Xu L, Zhu J, Zhang X, Yang Y, Song N. Immuno-isolation strategy with tacrolimus-loaded nanofilm promotes stable stem cell-based cartilage regeneration. Adv Funct Mater 2025;35:2414567.

