# Supplementary material for: Sustained in situ exosome delivery reprograms the diabetic wound microenvironment to unlock immuno-angiogenic repair
Source: Regen Biomater. 2026 Jul 13;13:rbag116. doi: 10.1093/rb/rbag116 (PMC13676320; doi:10.1093/rb/rbag116)
Supplement: Supplementary file 1 [file rbag116_supplementary_data.docx]

**Supporting Information**

**Sustained In Situ Exosome Delivery Reprograms the Diabetic Wound Microenvironment to Unlock Immuno-Angiogenic Repair**

*Ning Xu^1,2†^, Lili Wang^2†^, Yingliang Wei^3†^, Pingzhou Zou^2^, Suyang Xu^2^, Chenjun Liu^1^, Bohao Yin^1^, Junjie Guan^1^, Zhiyuan Fan^1^, Shengting Wu^1^, Huiyong Dai^1^, Sa Song^1*^, Xin Qi^1*^, Wei Zhang^1*^*

^1^ National Center for Orthopaedics, Shanghai Sixth People's Hospital, Shanghai 200233, China; Department of Orthopaedic Surgery, Shanghai Sixth People's Hospital Affiliated to Shanghai Jiao Tong University School of Medicine, Shanghai 200233, China;

^2^ Shanghai Eighth People's Hospital, Shanghai 200235, China;

^3^ Shanghai Normal University College of Life Science.

^†^ These authors contributed equally to the work.

*** Corresponding authors**: Dr. Wei Zhang, Email: [orthozhang@sjtu.edu.cn](mailto:orthozhang@sjtu.edu.cn); Dr. Xin Qi, Email: [qixin19871012@163.com](mailto:qixin19871012@163.com); Dr. Sa Song, Email: [songsa1971@163.com](mailto:songsa1971@163.com).

**Figure S1.** **Characterization of EE nanoparticles.** (A) Nanoparticle tracking analysis of EPC-Exos showing the size distribution and particle concentration. (B) Western blot analysis of the cellular marker Calnexin and exosomal surface markers (CD9, CD63, and CD81). (C) Release kinetics of exosomes from EE@PG nanofibrous scaffolds over a 21-day period.

**Figure S2. Released EPC-Exos collected at 72 h retained basic exosomal characteristics and pro-angiogenic activity.** (A) Western blot analysis of CD9 and CD63 expression in fresh EPC-Exos and released EPC-Exos collected from EE@PG at 72 h. (B) Representative tube formation images of HUVECs treated with fresh EPC-Exos or 72 h released EPC-Exos from EE@PG.

**Figure S3.** FT-IR spectrum of EE@PG nanofibrous mats.

**Figure S4.** Semi-quantitative ESR analysis of hydroxyl radical (•OH) levels in different groups (Control, PG, and EE@PG).

**Figure S5.** Semi-quantitative ESR analysis of superoxide anion (•O_2_^−^) levels in different groups (Control, PG, and EE@PG).

**Figure S6.** Semi-quantitative analysis of the percentages of live and dead cells derived from the live/dead staining images in **Figure 2C**.

**Figure S7.** Lysotracker staining of cells incubated with EE@PG-FITC at different time points (2, 4, and 6 h).

**Figure S8.** Semi-quantitative analysis of Lysotracker fluorescence in cells incubated with EE@PG-FITC at different time points (2, 4, and 6 h).

**Figure S9.** GO enrichment analysis of up-regulated and down-regulated genes in the EE@PG and Control groups (top enriched terms shown).

**Figure S10.** Body weight changes in different groups (Sham and EE@PG).

**Table S1. Primers included in this study**

| **Genes** | **Forward Primer (5’-3’)** | **Reverse Primer (5’-3’)** |
| --- | --- | --- |
| *c-MYC* | TTACAACACCCGAGCAAGGA | AAATACGGCTGCACCGAGTC |
| *ID1* | GGAATCCGAAGTTGGAACCC | CAGCGACACAAGATGCGATC |
| *COX2* | GATGATTGCCCGACTCCCTT | GAAAAGGCGCAGTTTACGCT |
| *VEGFA* | ATCACCATGCAGATTATGCGG | CCCCTTTCCCTTTCCTCGAAC |
| *GAPDH* | ATCCCATCACCATCTTCC | GAGTCCTTCCACGATACCA |

**Table S2. EPC-Exos loading efficiency of EE@PG**

| **Sample** | **Initial exosomes *M_0_* (μg)** | **Supernatant *M_s_* (μg)** | **Wash fraction *M_w_* (μg)** | **Loaded amount (μg)** | **Loading efficiency (%)** |
| --- | --- | --- | --- | --- | --- |
| 1 | 200.0 | 34.2 | 12.8 | 153.0 | 76.5 |
| 2 | 200.0 | 31.6 | 14.1 | 154.3 | 77.2 |
| 3 | 200.0 | 36.4 | 11.9 | 151.7 | 75.9 |
